# The Chemical Variability, Nutraceutical Value, and Food-Industry and Cosmetic Applications of Citrus Plants: A Critical Review

**DOI:** 10.3390/antiox12020481

**Published:** 2023-02-14

**Authors:** Anis Ben Hsouna, Carmen Sadaka, Ivana Generalić Mekinić, Stefania Garzoli, Jaroslava Švarc-Gajić, Francisca Rodrigues, Simone Morais, Manuela M. Moreira, Eduarda Ferreira, Giorgia Spigno, Tanja Brezo-Borjan, Boutheina Ben Akacha, Rania Ben Saad, Cristina Delerue-Matos, Wissem Mnif

**Affiliations:** 1 Laboratory of Biotechnology and Plant Improvement, Centre of Biotechnology of Sfax, B.P “1177”, Sfax 3018, Tunisia; 2 Department of Environmental Sciences and Nutrition, Higher Institute of Applied Sciences and Technology of Mahdia, University of Monastir, Monastir 5000, Tunisia; 3 Madison, WI 53704, USA; 4 Department of Food Technology and Biotechnology, Faculty of Chemistry and Technology, University of Split, R. Boškovića 35, HR-21000 Split, Croatia; 5 Department of Chemistry and Technologies of Drug, Sapienza University, P.le Aldo Moro 5, 00185 Rome, Italy; 6 Faculty of Technology, University of Novi Sad, Bulevar Cara Lazara 1, 21000 Novi Sad, Serbia; 7 REQUIMTE-LAQV, Instituto Superior de Engenharia do Porto, Rua Dr. António Bernardino de Almeida, 431, 4249-015 Porto, Portugal; 8 DiSTAS, Department for Sustainable Food Process, Università Cattolica del Sacro Cuore, 29122 Piacenza, Italy; 9 Department of Chemistry, Faculty of Sciences at Bisha, University of Bisha, P.O. Box 199, Bisha 61922, Saudi Arabia

**Keywords:** *Citrus*, orange peels, nutraceutical values, industrial applications, chemical composition

## Abstract

*Citrus* fruits occupy an important position in the context of the fruit trade, considering that both fresh fruits and processed products are produced on a large scale. Citrus fruits are recognized as an essential component of the human diet, thanks to their high content of beneficial nutrients such as vitamins, minerals, terpenes, flavonoids, coumarins and dietary fibers. Among these, a wide range of positive biological activities are attributed to terpenes and flavonoids derivatives. In this review, a list of bibliographic reports (from 2015 onwards) on the phytochemical composition, beneficial effects and potential applications of citrus fruits and their by-products is systematically summarized. In detail, information regarding the nutraceutical and medicinal value closely linked to the presence of numerous bioactive metabolites and their growing use in the food industry and food packaging, also considering any technological strategies such as encapsulation to guarantee their stability over time, were evaluated. In addition, since citrus fruit, as well as its by-products, are interesting alternatives for the reformulation of natural cosmetic products, the sector of the cosmetic industry is also explored. More in-depth knowledge of the latest information in this field will contribute to future conscious use of citrus fruits.

## 1. Introduction

The genus *Citrus* belongs to the angiosperm subfamily Aurantioideae of the family Rutaceae. Rutaceae are a family of flowering plants with about 160 genera. The most economically important members of the family are *Citrus*, which include the orange (*Citrus sinensis*), lemon (*Citrus limon*), grapefruit (*Citrus paradisi*) and lime (mainly *Citrus aurantifolia*). In general, *Citrus* represents one of the most important fruit crops in the world, and is grown mainly in tropical and subtropical climates of the world [1]. There are hundreds of different citrus cultivars. Some varieties were discovered accidentally from populations, while others resulted from planned hybridization of *Citrus* fruits. Due to the complex biology and wide geographic distribution, which also allows crossing between species, the taxonomy of the genus *Citrus* is not yet fully understood [2].

*Citrus* plants are typically evergreen trees or shrubs with glossy, oval-shaped leaves. The flowers are usually white, with five petals, and are highly fragrant. The fruits are a type of berry with the pulp divided into segments filled with tiny juice-filled vesicles. Finally, the peel, or rind, is leathery and full of oil glands.

In the past, citrus fruits were marketed and consumed exclusively as fresh fruit, as they retained their particular characteristics even after harvesting, which facilitated their marketing. Over time, the processing of fruit has become a necessity to meet increasingly diverse consumer demand [3]. Large-scale citrus processing began in the early 20th century with the spread of industrially produced citrus juices in some United States of America (USA) states, such as California and Florida [3]. It is estimated that the citrus-processing industry uses 33% of citrus for fruit-juice production as well as the production of jellies and jams [4].

The increase in world *Citrus* production has been relatively constant over the past twenty years. The latest United States Department of Agriculture (USDA) report [5] on world citrus markets and trade indicates that the global orange production for 2021/22 is expected to increase by 1.8 million tons over the previous year to 49.0 million tons, thanks to favorable weather conditions in Brazil and Turkey. Brazil is the leading producer country, followed by China, the European Union, Mexico and USA, with 16.91, 7.55, 6.10, 4.28 and 3.46 million tons, respectively. The report stated that most of the increased production is for Internal processing, with 12.28, 1.97, 1.70, 0.80 and 0.25 million tons processed in Brazil, USA, Mexico, the EU and China, respectively. This means that in Brazil, more than 70% of the production is destined for processing, in USA and Mexico about 50%, in the EU 13% and in China only 3%. According to these data, global orange-juice production is 1.64 million tons, of which 1.14, 0.19, 0.17, and 0.062 are produced in Brazil, USA, Mexico, and the EU, respectively. Similar data can be found for tangerines and mandarins, with a global fresh production of 37.21 million tons (with leading countries being China, the EU and Turkey, with 27, 3.16 and 1.80 million tons, respectively). In this case, only 1.34 million tons are destined for processing (3.6%). The global production of grapefruit is 6.96 million tons (of which 5.20 million tons is produced in China), with 6.7% (0.47 million tons) processed. Finally, the global production of lemons and limes is 9.75 million tons (the leading producers are Mexico, Argentina, the EU and Turkey, with 3.22, 1.90, 1.57 and 1.34 million tons, respectively) and, in this case, 2.58 million tons (26%) are processed (1.49 million tons are processed only in Argentina). In the Mediterranean basin, citrus fruits are mainly produced for the fresh-fruit market, with Spain being the main producer. Oranges account for most of the *Citrus* fruit, followed by mandarins, lemons, limes and grapefruits [6].

In recent decades, the production of mandarins has increased, to the detriment of fresh oranges. According to the Food and Agriculture Organization (FAO), the consumption of fresh oranges is decreasing in industrialized countries, while it is increasing in developing countries such as Mexico, India, Argentina, Brazil, and China. *Citrus* fruits are grown under very different climatic conditions, being exposed to abiotic stress factors such as soil acidity, increasing water scarcity in many countries and floods with waterlogging in others or even frost or excessive high temperatures. All these situations can lead to tree decline and, consequently, to a production decrease [7].

*Citrus* fruits have the unique value of containing essential nutrients, whose content varies according to ripeness [8]. Due to the high content of bioactive compounds in the different parts of the plant [9], they are used in various fields such as food, and the cosmetic and pharmaceutical industries as additives, spices, cosmetic ingredients and chemoprophylactic drugs [7]. Several in-vitro and in-vivo studies have reported that many citrus species affect the immune system, reproductive ability, and the cardiovascular and central nervous systems [10]. Citrus fruits have potential health benefits such as antimicrobial, anti-inflammatory, antiviral and anticancer properties, among others. Their beneficial health effects have been mainly attributed to secondary metabolites and, among these, carotenoids and flavonoids (especially the dominant class of flavanones) are particularly effective due to their potent antioxidant properties and the most important therapeutic effects [11].

In parallel with the large number of citrus fruits processed, large amounts of waste are generated [12]. The by-products are mainly solid and semi-solid residues, peels (albedo and flavedo), pulp and seeds, but also liquid residues from the processing plants [4]. In particular, citrus peels are rich in substances such as insoluble carbohydrates, organic acids, fatty acids, phytosterols, volatile compounds and polyphenols, without neglecting carotenoids and vitamins (ascorbic acid and B complex vitamins) [13]. Some of these bioactive compounds can be used in various fields, from pharmaceutical and nutraceutical industries to foods, healthy beverages, and cosmetics [14]. In addition, citrus by-products can be considered as an excellent source of nutrients in terms of developing a sustainable circular economy [15,16,17,18,19].

Based on the USDA report [5], it can be estimated that during the processing of almost 23 million tons of citrus fruit in 2021/22, more than 10 million tons of citrus waste were generated. If citrus peels remain unused, they become waste and are a potential source of environmental pollution. Orange peel has traditionally been dried and marketed as animal feed [20] or used in the food industry [21], especially oils, pectin, enzymes, single-cell proteins, natural antioxidants, ethanol, organic acids, and prebiotics [1]. Currently, the extraction of phenolic compounds from OP has attracted significant scientific interest [22] for use as a natural antioxidant in various applications, as well as the production and use of functional fibers [23].

In this review, the chemical composition of a large number of citrus species and their bio-functionalities are summarized in the context of potential applications in various sectors, from cosmetics to pharmacy, and the nutraceutical and food industries, in order to gain a better knowledge of this food with valuable beneficial properties.

## 2. The Chemical Profiles and Compositions of Citruses

The main chemical components of citrus fruits are water (85–90%), sugars, fibers, fats, vitamins (especially vitamin C), minerals, proteins, organic acids, pectins, and secondary metabolites, such as essential-oil components, phenolic compounds, carotenoids, alkaloids, limonoids, and coumarins, etc. [24,25,26]. The chemical composition of various citrus fruits is well-documented, not only for the whole fruit but also of its parts (pericarp, juice, pomace, seeds), flowers and leaves (Table 1).

Although most of the fruit produced is consumed fresh, the rest is usually processed into products such as juices, marmalades, jams, jellies, candied peel, and flavoring substances, etc. [6]. The amount of organic-waste materials in citrus processing is extremely high, ranging from 50 to 70% (*w*/*w*) depending on the cultivar, final product and processing technology used. In addition, their chemical composition is influenced by the cultivar, cultivation method, harvesting time, stage of ripeness (maturity), techniques used for juice extraction, storage period and fruit conditions, and extraction mode, among others [13,24,26,27,28].

In recent years, researchers worldwide have been focused on investigating different procedures and methods for the complete exploitation, valorization, and re-use of citrus-processing wastes. The main by-product (residue) of the citrus-processing industry is citrus peel, which is produced in large amounts in a short period of time due to the fruits’ seasonal production. Therefore, its disposal represents an important economic and environmental problem for producers [13]. On the other hand, it contains valuable and valued components with numerous beneficial biological properties, as it has been widely used in traditional medicine, food, and the cosmetic and pharmaceutical industries, as animal feed, organic soil conditioner or a substrate for compost production [25,29].

*Citrus* production is one of the world’s leading sectors of agricultural fruit production, with species such as oranges, lemons, limes, grapefruit, and tangerines having the greatest industrial importance. Table 1 provides a literature overview of the recent studies on the chemical composition of these major citrus cultivars, their plant parts, products and processing by-products.

**Table 1:** Overview of the studies (from 2015 to present) on the chemical constituents in major citrus species.

Plant Part (Origin) Variety	Isolate/Isolation Method	Major Chemical Components	Reference
Lemon (*C. limon* L.)			
Fruit (China)	EO, Hydrodistillation	(R)-(+)-Limonene (46%), Geranial (15.9%), Neral (10.6%), Citronellal (4.7%), α-Terpineol (4.0%), (−)-Isopulegol (3.9%), Linalool (2.3%).	[30]
Pulp (Indonesia)	Ethanolic, *n*-hexane, ethyl acetate extracts	Total phenolics (1.4–14.7 µg GAE/g), Flavonoids (8–30 µg QE/g), Luteolin-7-O-glucoside (0.024%), Rutin, Quercetin.	[31]
Peel (Tunisia) Beldi	Hydroethanolic extract	Total phenolics (105–204 mg GAE/g), Flavonoids (27–56 mg QE/g), Flavonols (9–26 mg RE/g), Condensed tannins (26–138 mg CE/g), Caffeoyl N-Tryptophan, Vicenin 2, Eriocitrin, Kaempferol-3-O-rutinoside, Quercetin-3-rutinoside,	[32]
Peel (Iraq)	Ethanolic, methanolic, ethyl acetate extracts	Coumarin, Ascorbic acid, Citric acid, Linoleic acid, Limonoid, Malic acid, D-Limonene, β-Carotene.	[29]
Peel (Indonesia)	Ethanolic, *n*-hexane, ethyl acetate extracts	Gallic acid (23.9 mg/L), 1,2-dihydroxybenzene (23.0 mg/L), Total phenolics (9–15.2 µg GAE/g), Flavonoids (25–29 µg QE/g).	[31]
Peel	EO, British Pharmacopoeia	D-Limonene (82.9%), β-Phellandrene (1.6%), β-Pinene (1.5%), γ-Terpinene (9.9%), β-Cymene (1.3%), α-Limonene diepoxide (1.2%).	[33]
Peel (Ethiopia)	EO, Clevenger-type apparatus	Limonene (49.7%), β-Pinene (17.1%), γ-Terpinene (7.5%), o-Cymene (2.2%), β-Bisabolene (2.4%), β-Caryophyllene (1.5%).	[34]
Peel (Nigeria) Osbeck	EO, Clevenger-type apparatus	Limonene (85.9%), Sabinene (3.9%), Myrcene (3.1%), Linalool (0.5%).	[35]
Peel (Iran)	EO, Clevenger-type apparatus	Limonene (61.4%), β-Pinene (13.1%), γ-Terpinene (11.3%), α-Pinene (2.4%), Sabinene (2.3%), Myrcene (1.6%), Geranial (1.5%), Neral (1.1%).	[36]
Peel (Algeria)	EO, Cold-pressing	Limonene (64.8%), γ-Terpinene (11.7%), β-Pinene (11.2%), α-Pinene (1.9%), β-Myrcene (1.7%), Geranial (1.7%), β-Bisabolen (1.0%).	[37]
Peel (Algeria) Eureka	EO, Clevenger-type apparatus	Limonene (61.3%), β-Pinene (9.7%), α-Citral (4.2%), γ-Terpinene (3.8%), *cis*-Citral (2.4%), β-Elemene (2.2%).	[38]
Peel (India) Burf	EO, Clevenger-type apparatus	Limonene (55.4%), Neral (10.4%), *trans*-Verbenol (6.4%), Decanal (3.3%), Ethyl cinnamate (2.2%), Ethyl *p*-methoxycinnamate (2.2%), *cis*-α-Bergamotene (1.6%), Geraniol (1.5%), *trans*-Carveol (1.3%), Nonanal (1.2%), Linalool (1.2%), α-Terpineol (1.1%).	[39]
Root (Cameroon)	Methanolic extract	Clausarin, Xanthyletin, Suberosin, E-suberenol, E-Methoxysuberenol, Thamnosmonin, Angelitriol, Hopey-hopin, Formlylumbelliferone, Atalantaflavone, Limonin, 1-(10–19) *abeo-7α*-acetoxy-10 β-hydroxyisoobacunoic acid-3,10-lactone.	[40]
Root (Cameroon)	EO, Hydrodistillation	Hexadecanoic acid, methyl ester (39.3%), β-Bisabolene (10.1%), (E)-9-octadecenoic acid, methyl ester (9.3%), α-Santalene (8%), Elemol (6.2%), (E)-5-Octadecene (6.1%), 1-Octadecene (5.7%).	[40]
Flowers (Tunisia) Osbeck	EO, Clevenger-type apparatus	Limonene (39.7%), β-Pinene (25.4%), α-Terpineol (7.3%), Nerolidol (6.9%), Farnesol (4.3%), Linalyl acetate (3.0%), Geranyl acetate (3.0%), Linalool (2.2%), Neryl acetate (1.7%).	[41]
Leaf (Cameroon)	Methanolic extract	Bergapten, 5-Hydroxy-6,7,8,4′-tetramethoxy flavone, 5-Hydroxy-6,7,8,3′,4′-pentamethoxy flavone, 5,4′-Dihydroxy-6,7,3′-trimethoxy flavone, 5,4′-Dihydroxy-6,7,8,3′-tetramethoxy flavone, 5,6,7,8,4′-Pentamethoxy flavone, 5-Hydroxynoracronicine, Asperfenamate, Stigmasterol, Sitosterol, Sitosterol-3-O-β-D-glucoside.	[40]
Leaf (Cameroon) Osbeck	Ethanolic, acetone, water extract	Alkaloids (12.2%), Saponins (5.5%), Total phenolics (208–289 mg GAE/g), Total flavonoids (447–1053 mg QE/g).	[42]
Leaf (Nigeria) Osbeck	EO, Clevenger-type apparatus	Limonene (31.5%), Sabinene (15.9%), Linalool (4.6%), (E)-β-Ocimene (3.9%), Myrcene (2.9%), α-Pinene (1.2%).	[35]
Leaf (Iran)	EO, Clevenger-type apparatus	Linalool (30.6%), Geraniol (15.9%), α-Terpineol (14.5%), Linalyl acetate (13.8%), Geranyl acetate (6.7%), β-Pinene (4.5%), Neryl acetate (4.2%).	[43]
Leaf (China)	EO, Hydrodistillation	Citronellal (75.3%), (R)-(+)-limonene (11.4%), Citronellol (6.7%), Citronellyl acetate (1.7%).	[30]
Orange (*C. sinensis* L.)			
Pulp (Spain) Osbeck	Juice, Mechanical squeezing, HS-SPME	1-Octanol (49–151), α-Pinene (28–65), β-Mircene (693–1340), Limonene (4310–5210), α-Terpinolene (54–106), Linalool (116–166), Valencene (698–1200)- units are ion peak areas divided by 10^6^.	[44]
Pulp (China) Newhall navel	Juice, Mechanical squeezing	Sucrose (53.4–67.5 g/L), Glucose (25.7–29.5 g/L), Fructose (23.1-25.3 g/L), Total phenolics (4.3–5.1 mmol GAE/L), Total flavonoids (1.9–2.3 mmol/L), Vitamin C (0.51–0.63 mg/g), Hesperidin (478–839 mg/L), Narirutin (249–295 mg/L), Limonin (3.4–14.0 mg/L).	[45]
Pulp (Italy) Osbeck	Juice, Mechanical squeezing	Lucenin-2 (4.5–7.2 mg/L), Vicenin-2 (32.2–36.2 mg/L), Stellarin-2 (0.8–6.5 mg/L), Narirutin 4′-*O*-glucoside (0.5–10.1 mg/L), Quercetin-3-*O*-hexoside (0.7–4.1 mg/L), Narirutin (14.4–61.3 mg/L), Hesperidin (106–426 mg/L).	[46]
Pulp (Florida) Valencia	Juice, Mechanical squeezing	Insoluble solids (14–18 mg/g), Soluble solids (101–136 mg/g), Pectine (0.04–0.56 mg/g), Titrable acids (5.7–10.7 mg/g), Citric acid (6–1060 mg/g), Malic acid (2 mg/g), Sucrose (48–66 mg/g), Glucose (10–40 mg/g), Fructose (19–37 mg/g), Limonin (0.5–5.6 µg/g), D-Limonene (58–336 mg/L), Valencene (2.5–2.7 mg/L), Linalool (1.7–3.2 mg/L), Myrcene (2.6 mg/L), Acetaldehyde (4.9–8.1 mg/L), Ethyl acetate (3.3–3.9 mg/L).	[47]
Pulp (China) Osbeck (Tarocco blood oranges)	Juice, Mechanical squeezing	Total anthocyanine content (55–109 µg/g) (Delphinidin 3-glucoside, Cyanidin 3- galactoside, Cyanidin 3-glucoside, Delphynidin 3-(6″-malonylglucoside), Cyanidin 3-(3″-O-β-glucopyranosyl-6″-O-malonyl-β-glucopyranoside), Cyanidin 3-(6″-malonylglucoside, Cyanidin 3-(6″-dioxalylglucoside), Delphynidin-3-rutinoside, Cyanidin malonyl-(dioxalyl)-hexoside); D-limonene, Aromandendrene, Linalool, β-myrcene, D-carvone, ethyl butanoate.	[48]
Pulp (Mexico)	Methanolic extracts	Total phenolics (6.46 mg/g); Ferulic acid-*O*-hexoside (157.6 μg/g), Sinapic acid (414.7 μg/g), Isosakuranetin-7-*O*-rutinoside (428.8 μg/g), Naringenin-7-*O*-rutinoside (647.8 μg/g), Naringen-7-*O*-neohesperidoside (1428.8 μg/g), Naringenin-7-*O*-rutinoside-4′-*O*-glucoside (41.9 μg/g), Hesperetin-7-*O*-rutinoside (4434.9 μg/g), Apigenin-6,8-di-*C*-glucoside (216.8 μg/g), Vitexin-2″-*O*-xiloside (199.4 μg/g).	[49]
Peel (Egypt)	Water and ethanolic extracts	Narirutin (29 μg/g), Naringin (27 μg/g), Hesperetin (17 μg/g), Hesperetin-7-*O*-rutinoside naringenin (15 μg/g), Quinic acid (13 μg/g), Datiscetin-3-*O*-rutinoside (11 μg/g), Sakuranetin (9 μg/g).	[50]
Peel (Nigeria) Navel	Decoct	Quercitrin (22.6 mg/g), Rutin (17.9 mg/g), Quercetin (14.0 mg/g), Catechin (12.5 mg/g), Epicatechin (6.1 mg/g), Luteolin (5.9 mg/g), Naringin (5.7 mg/g), Kaempferol (3.8 mg/g), Caffeic acid (3.6 mg/g).	[51]
Peel (China) Osbeck Newhall	Ethanolic and ethyl acetate extracts	Sinensetin (67.3 μg/mg), Narirutin (55.6 μg/mg), Nobiletn (37.0 μg/mg), Hesperidin (31.5 μg/mg), 4′,5,6,7-Tetramethoxyflavone (22.5 μg/mg), 3,3′,4′,5,6,7-Hexamethoxyflavone (17.7 μg/mg), Didymin (12.5 μg/mg), 3,3′,4′,5,6,7,8-Heptamethoxyflavone (12.2 μg/mg).	[45]
Peel (China)	Methanolic extract, UAE	Isosinensetin (21.6–63.9 μg/g), Sinensetin (0.33–0.89 mg/g), 5,6,7,4′-Tetramethoxyflavone (8.6–21.7 μg/g), Nobiletin (0.42–1.01 mg/g), 5,7,8,4′-Tetramethoxyflavone (0.08–0.25 mg/g), 3,5,6,7,8,3′4′-Heptamethoxyflavone (0.15–0.35 mg/g), Tangeritin (0.07–0.15 mg/g), 5-hydroxy-6,7,8,3′,4′-Pentamethoxyflavone (11.3–46.7 μg/g), Gardenin A (2.9–19.0 μg/g).	[52]
Peel (USA)	Methanolic extract	*p*-Coumaric acid (18 µg/g), Ferulic acid (19 µg/g), Narirutin (1.34 mg/g), Hesperidin (16.3 mg/g).	[53]
Peel (China) Osbeck Brocade	Methanolic, DMSO, water extracts	Phenolic acids (Ferulic, *p*-Coumaric, Sinapic, Caffeic acid, Syringic, Vanillic, *p*-Hydroxybenzoic, Benzoic), Flavanones (Hesperetin, Hesperidin, Neohesperidin, Naringenin, Naringin, Didymin), Flavonols (Quercetin, Rutin), Flavones (Rhoifolin, Apiin, Luteolin), Polymethoxyflavones (Sinensetin, Tangeretin, Nobiletin).	[54]
Peel (Mexico) Valencia	Ionic Liquid-based MAE	Limonene (84.6–95.7%), 1-r-α-Pinene (0.2–1.9%), Linalol (0.9–1.3%), Eugenol methyl ether (0.2–1.5%), Eugenol (2.0%), Linalyl formate (1.5%), 1,2-Benzenedicarboxilic acid Mono(2-Ethylhexyl) ester (5.4%).	[55]
Peel (Ethiopia)	EO, Clevenger-type apparatus	Limonene (95.2%), N-methyl-1,3-propanediamine (2.9%), β-Myrcene (1.1%), 3-Carene (0.8%).	[34]
Peel (Pakistan) Mussami	EO, Clevenger-type apparatus	Limonene (48.9%), Geraniol (10.0%), Citraniol (10.1%), Eugenol (7.5%).	[56]
Peel (Pakistan) Red blood orange	EO, Clevenger-type apparatus	Limonene (46.3%), Geraniol (24%), Eugenol (12.9%), Citraniol (10.4%).	[56]
Peel (Iran)	EO, Clevenger-type apparatus	Limonene (90.5%), *trans*-Carveol (1.1%), Carvone (1.1%), *cis*-Linalool oxide (1.0%), β-Myrcene (0.9%).	[57]
Peel (Morocco) Navel	EO, MAHD	D-Limonene (89.9%), α-Sinensal (2.7%), β-Myrcene (2.3%), Capric aldehyde (1.9%), Linalool (1.2%), β-Pinene (0.7%), δ-Terpinene (0.6%).	[58]
Peel (Morocco) Navel	EO, Clevenger-type apparatus	D-Limonene (92.7%), α-Bergamotene (2.7%), β-Myrcene (2.2%), Sabinen (0.8%), (+)-Carene (0.8%), Capric aldehyde (0.7%), δ-Terpinene (0.6%), α-Pinene (0.6%), Linalool (0.2%).	[58]
Peel (Nigeria) Osbeck	EO, Clevenger-type apparatus	Limonene (92.1%), β-Myrcene (2.7%).	[59]
Seed (Nigeria)	Oil, Soxhlet Extraction	Total lipids (34.5%), Total saturate acids (28.5%), Total unsaturated acids (71.5%), Monounsaturated acid (29.7%), Polyunsaturated acids (41.8%); Linoleic acid (36.2%), Oleic acid (27.4%), Palmitic acid (21.1%), Stearic acid (4.8%), α-Linolenic acid (3.5%).	[60]
Leaf (Vietnam) Osbeck	EO, Clevenger-type apparatus	β-Pinene (16.9%), Limonene (13.8%), β-Ocimene (7.5%), Terpinen-4-ol (5.7%), Linalool (5.2%), β-Cubebene, (4.9%), Sabinene (4.7%), Nerol (3.8%), Geraniol (2.7%).	[61]
Leaf (Egypt) Navel cultivars	EO, Clevenger-type apparatus	Sabinene (8.3–28.8%), 2-Carene (11.3–16.7%), *cis*-β-Ocimene (10.2–13.9%), D-Limonene (6.5–12.0%), γ-Terpinene (2.0–4.5%), β-Citronellal (0.3–7.7%), Terpinen-4-ol (3.0–6.6%), β-Myrcene (3.4–5.6%), Linalool (0.2–5.3%), β-Elemene (2.6–14.2%).	[62]
Lime (*C. aurantiifolia*)			
Pulp (Nigeria)	Juice	Flavonoids (7.1 mg/g), Tannins (5.3 mg/g), Phenolics (0.7 mg/g), Terpenes (0.6 mg/g).	[63]
Pulp (Iran)	EO, Clevenger-type apparatus, Extract, Static headspace	Limonene (49.3–71.7%), β-Pinene (8.5–21.7%), γ-Terpinene (7.3–9.0%), Myrcene (1.8%), α-Pinene (1.7–6.8%%), *trans*-Ferulic acid (2.3–2.8 mg/g), Hesperidin (0.3–2.1 mg/g), Ellagic acid (0.2–1.8 mg/g), Quercetin (0.03–0.8 mg/g), Rosmarinic acid, Hesperetin, Gallic acid, Catechin, Chlorogenic acid, *p*-Coumaric acid, Vanillin.	[64]
Peel (India)	EO, Clevenger-type apparatus	Palatinol-1C (13.3%), Limonene (12.9%), Carvon (9.1%), 2-Isopropenyl-5-methyl-4- hexanal (5.5%), *cis*-Cavacrol (5.3%).	[65]
Peel (Vietnam)	EO, Clevenger-type apparatus	Limonene (62.2%), γ-Terpinene (12.4%), β-Pinene(11.7%), β-Cymene (2.8%), 1R-α-Pinene (2.2%), Sabinene (1.5%).	[66]
Peel (Iran)	EO, Clevenger-type apparatus	Limonene (40.3%), β-Pinene (9.5%), α-Terpineol (10.9%), γ-Terpinolene (8.9%).	[57]
Peel (Italy)	Cold pressing	α-Phellandrene (48.5%), *p*-Cymene (16.5%), α-Pinene (12.6%), (E,E)-α-Farnesene (12.6%).	[67]
Peel (Vietnam)	EO, Clevenger-type apparatus, MAHD	Limonene (65.9 and 71.9%), α-Pinene (1.9 and 0.8%), β-Pinene (11.3 and 5.2%), β-Cymene (1.5 and 13.8%), α-Bergamotene (1.2 and 1.3%), Sabinene (1.5 and 1.6%).	[68]
Peel, Leaf (Brazil)	EO, Clevenger-type apparatus	Limonene (32.7–77.5%), Linalool (3.5–20.1%), Citronellal (3.2–14.5%), Citronellol (2.0–14.2%).	[69]
Leaf (India)	EO, Clevenger-type apparatus	Citral (13.5%), Limonene (11.6%), 1,2- Cyclohexanediol, 1-methyl-4-(1-methylethenyl) (11.3%), Geraniol (10.6%), Decanol (6.2%).	[65]
Leaf (Vietnam)	EO, Clevenger-type apparatus	Limonene (30.1%), β-Pinene (19.3%), Citronellol (3.9%), β-Caryophyllene (3.6%), β-Ocimene (3.5%), α-Terpineol (3.1%).	[61]
Leaf (Oman)	EO, Clevenger-type apparatus	D-Limonene (63.4%), 3,7-Dimethyl-2,6-octadien-1-ol (7.1%), Geraniol (6.2%), E-Citral (4.4%), Z-Citral (3.3%), β-Ocimene (2.3%).	[70]
Leaf (Nigeria)	EO, Clevenger-type apparatus	D-Limonene (57.8%), Neral (7.8%), Linalool (4.8%), Sulcatone (3.5%), Isogeraniol (3.5%).	[71]
Leaf (Benin)	Aqueous and ethanolic extracts	Phenolics (250–350 µg GAE/g), Flavonoids (6–24 µg RE/g).	[72]
Tangerine (*C. reticulata*)			
Pulp (China)	Cyclodextrine-based liquid-phase pulsed discharge extraction	Total flavonoids (17.8 mg/g), Narirutin (4.2 mg/g), Hesperidin (15.7 mg/g), Nobiletin (0.6 mg/g), Tangeretin (0.5 mg/g).	[73]
Peel (China) Dahongpao	Methanolic extract	Tricin, Naringenin-7-*O*-glucoside (Prunin), Apigenin, Xanthohumol, Epicatechin gallate, Curcumin, Dihydromyricetin, Hesperetin 7-rutinoside (Hesperidin), Isoschaftoside, Astilbin, Vicenin-3, Eriocitrin, 6-Gingerol, Tectorigenin, Phloridzin, Naringenin 7-*O*-neohesperidoside (Naringin), Kaempferol 3-*O*-rutinoside (Nicotiflorin), Acacetin, Troxerutin (Trihydroxyethyl rutin), Quercetin 3-*O*-glucoside (Isotrifoliin), Biochanin A, Prunetin, Narirutin, Isoquercitroside, Theaflavin, Diosmin.	[74]
Peel (Portugal) Blanco	Aqueous and hydroethanolic extracts, SFE	Naringenin, Quercetin, Hesperidin, Naringin, Tangeretin, Rutin, Chlorogenic acid, Caffeic acid, Ferulic acid, Hesperitin.	[75]
Peel, Flesh, Seed (China) Blanco cv. Chachiensis (Chachi)	Acetone extracts	Peel: Naringin (555–581 µg/g), Hesperidin (3771–7491 µg/g), Nobiletin (1695–2011 µg/g), Tangeretin (597–646 µg/g), Chlorogenic acid (410–553 µg/g), Ferulic acid (270–356 µg/g). Flesh: Naringin (24.8–31.0 µg/g), Hesperidin (896–1076 µg/g), Neohesperidin (17.5–27.2 µg/g), Nobiletin (15.5–22.5 µg/g), Chlorogenic acid (79–99 µg/g), Caffeic acid (87–138 µg/g). Seed: Naringin (16.5–29.2 µg/g), Hesperidin (249–334 µg/g), Neohesperidin (20.2–56.3 µg/g), Ferulic acid (83.8–90.2 µg/g).	[76]
Peel (China) Blanco	EO, Hydrodistillation	D-Limonene (88.4%), γ-Terpinene (4.8%), Geranyl acetate, β-Elemen, δ-Elemen, Cyclohexane, 2,4-Diisopropenyl-1-methyl-1-vinyl, Gemacrene B, γ-Elemen, Neryl acetate, (-)-Spathulenol.	[77]
Peel (Brazil) Blanco	EO, Clevenger-type apparatus	Limonene (80.2%), Myrcene (6.7%), Linalool (3.7%), Sabinene (2.6%), α-Pinene (2.1%), ρ-Mentha-2,4(8) diene (1.5%), ρ-Mentha-1 (7),8-diene (0.7%), *n*-Decanal (0.5%), Terpinen-4-ol (0.3%), α-Terpineol (0.3%).	[78]
Peel (China) Chachi	EO, Hydrodistillation	D-Limonene (75.1%), γ-Terpinene (13.5%), Methyl methanthranilate, α-Sinensal, Champhene, Thymol, Citronellal, Perilla aldehyde, (R)-(+)-β-Citronellol.	[77]
Peel (Pakistan) Kinnow	EO, Clevenger-type apparatus	Limonene (54.6%), Citraniol (14%), Geraniol (12.7%), Eugenol (8.9%).	[56]
Peel	EO, British Pharmacopoeia.	D-Limonen (84.9%), δ-3-Carene (3.1%), β-Cymene (2.1%), β-Pinene (1.0%).	[33]
Peel (China) Ponkan	EO, Clevenger-type apparatus	Limonene (72.5%), γ-Terpinene (11.2%), β-Myrcene (3.0%), α-Pinene (1.3%), Linalool (1.9%), Octanal (0.6%), β-Pinene (0.6%), α-Terpinlene (0.5%), Sabinene (0.3%), β-Decanal (0.2%).	[79]
Grapefruit (*C. paradisi*)			
Fruit, Juice-processing residues (Spain)	Extract, Steam explosion	D-limonene (87.1–93.7%), β-Myrcene (1.4–2.4%), Carvone (0.02–1.6%), (E)-Caryophyllene (0.4–1.5%), Pectic hydrocolloids (11–27 mg/g), Naringin (12–67 µg/g), Narirutin (4.1–7.9 µg/g), Naringin-4′-O-glucoside, Hesperidin glucoside.	[27]
Fruit (Iran)	Volatiles, Headspace single-drop microextraction	D-Limonene, β-Myrcene, α–Pinene, β-Pinene.	[80]
Pulp (Italy) Marsh, Star Ruby	Juice, Mechanical squeezing	Total Soluble Solids (10.8–13.4%),Total Acidity (0.6–1.0 mg citric acid/100 mL), Total phenolics (153–167 mg GAE/L), Total flavonoids (310–390 mg QE/L), Naringin (198–288 mg/L), Ascorbic acid (455–680 mg/L), Narirutin (37–39 mg/L), Poncirin (14–17 mg/L), Flavanones (narirutin, naringin, hesperidin, neohesperidin, and poncirin), flavones (rutin) and aglycones (quercetin, naringenin and hesperetin).	[81]
Pulp (Egypt)	Juice, Mechanical squeezing	Total soluble solids (11.6–12%),Total acidity (11.3–19.2 mg citric acid/L), Minerals (mg/L): P (890–930), Mg (150–340), Ca (210–480), K (1–6), Na (580–720), Fe (200–390), Cu (0.3–0.9), Zn (2–4), Mn (150–340), Total carbohydrates (8.1–85 g/L), Total fiber (44–66 mg/g), Total phenolics (7760–14080 mg GAE/L), Total flavonoids (195–251 mg CE/L), Total carotenoids (0.01–0.48 mg/L), Ascorbic acid (388–392 mg/L), Thiamine (5–6.5 mg/L), Riboflavin (0.4–0.5 mg/L).	[82]
Pulp (Spain) Star Ruby	Juice, Extract liquidization, Spray-drying, Oxalic acid and methanolic extracts	Total phenolics (12.7–12.9 mg GAE/g), Total flavonoids (43.1–65.9 mg QE/g), Delphinidin-3-glucoside, Hesperitin-7-O-glucoside, Hesperidin, Neohesperidin.	[83]
Pulp (Spain) Star Ruby	Juice, Extract, Freeze-drying, Spray-drying	α-Tocopherol (6–10 µg/g), Ascorbic acid (3.2–3.8 mg/g), Total phenolics (4.99–10.04 mg/g), Total phenolic acids (0.07–0.15 mg/g), Total flavonoids (4.9–9.9 mg/g), Narirutin (0.74–1.42 mg/g), Naringin (3.31–6.81 mg/g), Poncirin (0.28–0.48 mg/g).	[84]
Pulp (India)	Juice, Mechanical squeezing	Total soluble solids (10.3–12.4 °Brix), Acidity (1.2–2.0 g citric acid/kg), 1-(3-Ethyloxiranyl)-ethanone (up to 29%), 3-Hexen-2-one (9.9–11.6%), Limonene (0.7–15.4%).	[85]
Pulp (Pakistan) Shamber Tarnab	Juice	Total soluble solids (7.9 °Brix), Titrible acidity (1.4%), Ascorbic acid (~0.45 mg/g).	[28]
Peel (Sudan)	-	Ash (1.5–1.6%), Protein (1.1–1.2%), Oil (0.2–0.4%), Fiber (1.7–1.8%), Alcohol insoluble solids (9.5–10.5%), Titrable acidity (0.16–0.22%), Ascorbic acid (0.15–0.16%), Reducing sugars (10.2–10.4%), Total sugars (18.9–19.8%), Calcium (6.9–7.1 µg/g), Magnesium (1.7 µg/g), Total pectin (25%).	[86]
Peel (Spain)	Extract, ASE	Total phenolics (28–85 mg GAE/g), Naringin (43.5–160.1 mg/g), Naringenin (2.4–8.5 mg/g), Isonaringin (3.6–13.4 mg/g).	[87]
Peel (Egypt)	-	Proteins (64 µg/g), Fats (38 µg/g), Fibers (28 µg/g), Ash (82 µg/100 g), Carbohydrates (0.79 mg/g), Lycopene (0.43 mg/g), Ascorbic acid (0.52 mg/g), Total phenolics (10.78 mg GAE/g), Flavonoids (1.74 mg CE/g).	[26]
Peel (Argentina)	EO, Cold-pressing, Steam distillation	Limonene (87.9–88.5%), Myrcene (2.8–3.5%), β-pinene (1.2%), γ-Terpinene (1.1%).	[88]
Peel (Algeria)	EO, MAHD, Hydrodistillation	Limonene (85.5–87.5%), β-Myrcene (3.0–3.2%), Nootkatone (1.8%).	[89]
Peel (South Africa)	EO, Clevenger-type apparatus	D-limonene (87–90%), β-Myrcene (2–4%), γ-Terpinene (0.05–2%).	[90]
Peel (India)	EO, Clevenger-type apparatus	1-Methyl-4-(1-methylethenyl)-cyclohexene (up to 84.3%), Myrcene (4.0–6.2%), 2,6,6-Trimethyl-bicyclo [3.1.1] hept-2-ene (1.0–1.6%)	[85]
Peel (Pakistan)	EO, Clevenger-type apparatus	Total phenolics (121 mg GAE/L), Flavonoids (76 mg CE/L), Triacetin (53.5%), Di-*n*-octyl-phthalate (17.3%), Octanal (9.2%), D-Limonene (9.2%), Alkaloids, Saponins.	[91]
Peel	EO, British Pharmacopoeia	D-Limonene (91.8%), δ-3-Carene (2.07%), β-Pinene (1.1%).	[33]
Peel (China)	EO, Molecular distillation	Limonene (93.3%), β-Myrcene (2.2%), α-Pinene (0.8%), Sabinene (0.6%), *cis*-Limonene oxide (0.4%), Carvone (0.4%), Octanal (0.4%), *trans*-Limonene oxide (0.3%).	[92]
Leaf (South Africa)	EO, Clevenger-type apparatus	β-Phellandrene (90–91%), Furanoid (0.6–2%), Caryophyllene (0.08–2%).	[90]
Pomelo (*C. grandis*)			
Pulp (India)	Juice, Mechanical squeezing	Total phenolics (1834 mg GAE/L), Total flavonoids (529 mg QE/L), Fructose (12 g/L), Glucose (11 g/L), Sucrose (50 g/L), Citric acid (12 g/L), Malic acid (1.5 g/L), Tartaric acid (0.13 g/L), Succinic acid (0.22 g/L), Ascorbic acid (0.32 g/L), (R)-Limonene (1.67 mg/g), Octanal (13 µg/g), Linalool (21 µg/g), Ethyl butanoate (107 µg/g), Terpineol (13 µg/g), Citral (16 µg/g), α-Pinene (21 µg/g), Ethyl butyrate (1.02 mg/g), 2-Phenylethanol (1736 µg/g).	[93]
Pulp (China) Shatianyu, Lingpingyu Guangximiyu-R, Guangximiyu-W, Yuhuanyu	Acetone extracts	Total phenolics (0.92–1.71 mg GAE/g), Total flavonoids (0.13–1.93 mg CE/g); Cigranoside A (0.17–11.45 μg/g), Cigranoside B (0.19–17.73 μg/g), Cigranoside C (1.88–33.75 μg/g), Cigranoside D (0.69–38.0 μg/g), Cigranoside E (0.22–20.3 μg/g), Bergamjuicin (0.1–51.5 μg/g), Neoeriocitrin (0.8–14.7 μg/g), Melitidin (19.4–233.4 μg/g), Rhoifolin (1.64–4.14 μg/g), Naringin (24.5–301 μg/g), Hesperidin (0.004–0.028 μg/g), Isoquercitrin (0.1–1.09 μg/g).	[92]
Peel (Korea) Osbeck	Ethanolic extract (dichloromethane fraction)	Naringin (0.3 mg/g), Narirutin (0.3 mg/g), Neohesperidin (2.0 mg/g), Hesperidin (0.4 mg/g), Rutin (0.2 mg/g), Apigenin (1.0 mg/g), Hesperetin (0.8 mg/g), Isorhamnetin (1.4 mg/g), Kaempferol (1.4 mg/g), Luteolin (0.5 mg/g), Myricetin (1.3 mg/g), Naringenin (0.2 mg/g), Quercetin (0.6 mg/g), Rhaemnetin (1.9 mg/g), Taxifolin (7.0 mg/g), Nobiletin (70.5 mg/g), Sinensetin (76.2 mg/g), Tangeretin (14.1 mg/g).	[94]
Flavedo, albedo, juice sacs (China) Baishi, Cuixiangtian, Guanxi	Ethanolic extract, UAE	Limonin, Nomilin, Limonin glucoside.	[95]
Peel (Vietnam)	EO, Clevenger-type apparatus, Co-extraction, using citric acid	Limonene (87.9%), β-Pinene (2.7%), α-Phellandrene (1.3%), γ-Terpinene (0.5%), Linalool (0.26%), *trans*-β-ocimene (0.24%), *trans*-linalool oxide (0.18%), α-Terpinene (0.16%), *cis*-linalool oxide (0.12%), β-Citronellol (0.09%), *trans*-*p*-mentha-2,8-dien-1-ol (0.08%). Pectines (24%).	[96]
Peel (Vietnam)	EO, Hydrodistillation	Limonene (97.4%), β-Myrcene (1.2%), α-Phellandrene (0.7%), α-Pinene (0.5%), Sabinene (0.13%), β-Pinene (0.07%).	[97]
Peel (China)	EO, Steam distillation	D-Limonene (53.6%), Ocimene (4.4%), γ-Terpinene (1.6%), Myrcene (1.4%), α-Pinene (1.0%), β-Pinene (0.5%), Linalool (0.2%).	[98]
Leaf (Vietnam) Osbek	EO, Clevenger-type apparatus	Limonene (21.9%), Geraniol (10.7%), Nerol (10.4%), β-Caryophyllene (6.8%), β-Ocimene (6.4%), α-Phellandrene (4.0%), Citronellol (3.2%).	[61]

EO—essential oil, MAHD—microwave-assisted hydrodistillation, MAE—microwave-assisted extraction, UAE—ultrasonic-assisted extraction, ASE—accelerated solvent extraction, SFE—solid-phase extraction, HS-SPME—headspace solid-phase microextraction, GAE—gallic-acid equivalents, QE—quercetin equivalents, CE—catechin equivalents, RE—rutin equivalents.

## 3. Nutraceutical Value of Citrus

The nutraceutical and medicinal value of citrus fruits is mainly attributed to their richness in dietary fiber and bioactive compounds such as citric acid, polyphenols, terpenoids, vitamins, minerals, and essential oils. Their diverse phytochemical profile gives them high potential for the prevention and treatment of various diseases. Although citrus-plant extracts are widely used in the food, cosmetic, and nutraceutical industries, their pharmacological potential still needs to be confirmed by clinical trials.

### 3.1. Dietary Fibers

*Citrus* fruits contain soluble (such as pectin, fructans, and psyllium) and insoluble (such as cellulose, hemicellulose and lignin) fibers in varying amount in peel, pulp, and juices [99]. Soluble fibers can resist digestion in the small intestine, being fermented into a gel-like substance by the microflora in the large intestine, whereas insoluble fibers keep their structure intact, move through the gastrointestinal tract, and prevent constipation problems by softening the stool [100].

The consumption of dietary fiber has been shown to regulate physiological functions, and is linked with a lower risk of cancer, cardiovascular diseases, diabetes, obesity, and gastrointestinal disorders [101,102]. Furthermore, in a randomized cross-over clinical trial in women, consumption of dietary-fiber concentrates from citrus fruits over a 4-week period was shown to reduce both total serum cholesterol levels and high-density lipoprotein cholesterol levels [103]. Effective fiber intake can be obtained from natural dietary sources such as fruits, especially citrus fruits, vegetables, and some cereals [103]. However, in certain groups of people, such as the elderly, those with low dietary intake and those suffering from gastrointestinal disorders, or diagnosed with Parkinson’s disease, it is difficult to rely solely on dietary sources as the only source of fibers. Therefore, fiber supplements, including those containing fibers derived from citrus fruits, are used and available as over-the-counter capsules and powders. In addition, some citrus-fiber supplements can be added to low-fiber and fiber-free foods (e.g., commercial snacks, baked goods, and bread) without affecting the sensory properties of the food in question.

### 3.2. Citric Acid

The sugar/acid ratio is the main indicator of the quality and ripeness (maturity) of citrus fruits [104]. *Citrus* fruits contain mainly citric, malic, succinic, tartaric, and oxalic acids, with citric and malic acids being the most important constituents [104,105]. The commercial use of citric acid has led to an increasing demand for it. Citric acid is widely used in the food, beverage, pharmaceutical, nutraceutical, and cosmetic industries as an acidulant, preservative, emulsifier, sequestrant, flavoring and buffering agent [106]. Citric acid can be produced through fermentation from yeast and molds, while natural citric acid is obtained from citrus fruits [107]. Since citric acid is abundant in citrus fruits, especially in *C. aurantium* from bitter oranges, it can be used as a commercial substitute source for citric-acid production [104,105].

### 3.3. Polyphenolics: Flavonoids

Polyphenolics are the most abundant bioactive constituents of citrus fruits, but their concentration varies among citrus species, and this variation is also influenced by environmental conditions [108]. Polyphenolics can be divided into diferuloylmethanes, stilbenes, flavonoids, phenolic acids, and tannins [109]. The most important class of polyphenolics is flavonoids, which are divided into several subgroups, including chalcones, flavones, flavanones, flavonols, and isoflavones [110]. These subgroups have different dietary sources. *Citrus* fruits such as oranges, bergamots, lemons, and grapefruit are an important source of flavanones [110]. Like most monomeric flavonoids in nature, citrus flavanones occur as glycosides bound to various sugars. Flavonoid glycosides are distributed in different parts of citrus, but the largest amount is found in the solid parts: flavedo, albedo, and membranes [111].

It is well-known that phenolic acids and flavonoids are great antioxidants. Recently, the use of polyphenolics as natural antioxidants in foods to prevent lipid oxidation and increase the nutritional value of foods has attracted great interest [108]. In addition to their antioxidant potential, numerous studies suggest that citrus polyphenolics have antiviral, antimicrobial, antiallergenic, anti-ageing, anticarcinogenic, antidiabetic, cardioprotective and neuroprotective potential [6,53,111,112,113,114,115,116]. Polyphenolics from citrus also have anti-inflammatory effects by interacting with the nucleotide binding sites of regulatory enzymes that play a key role in the cellular inflammatory process, including receptor binding and cellular activity during inflammation. These compounds have been shown to regulate signaling at the molecular level, giving them the potential to prevent and treat diseases such as cancer, diabetes, neurodegenerative and cardiovascular diseases, and ageing [108,112,117,118,119]. In addition, citrus polyphenolics have prebiotic potential, especially for metabolic diseases [120], and improve blood glucose levels and lipid profiles. A small proportion of deglycosylated flavonoids are taken up by intestinal bacteria, while most of them are degraded to short-chain fatty acids, which supports microbiota homeostasis, among other beneficial effects on the human metabolism [121]. In addition, citrus flavonoids appear to improve mitochondrial dysfunction and lead to a reduction in acetylcholinesterase activity [122]. Hesperidin inhibits pleurisy, hesperetin limits increases in triacylglycerol and cholesterol levels in the liver, naringenin decreases plasma cholesterol levels and stimulates DNA repair after oxidative damage in human-prostate cancer cells, and naringin increases superoxide dismutase and catalase activity, thus playing an important role in regulating antioxidant capacity. Furthermore, naringin blocks H_2_O_2_-induced cytotoxicity and apoptosis and may affect H_2_O_2_-induced expression of apoptosis-associated genes or proteins [111]. The neuroprotective and neuromodulatory effects of citrus polyphenolics such as hesperetin, naringenin and their in-vivo metabolites can be explained by their ability to cross the blood–brain barrier [123]. Given the numerous examples of their health-promoting effects in the scientific literature, the citrus polyphenolics are valued for their considerable nutraceutical value [117], and their use represents a potentially useful approach for the prevention and treatment of a wide range of diseases. Although some clinical and preclinical studies have been conducted, much experimental and human research data are still needed to apply citrus polyphenolics in human medicine.

One of the main problems of flavonoids that affect their nutraceutical efficacy is their low bioavailability. This is due to their solubility in *citrus* juice [117], differences in preparation techniques (fresh/natural vs. preserved/commercial) [124], flavonoid metabolism and the gut microbiota responsible for this metabolism [117]. To increase their bioavailability, some citrus products, such as fruit juice, are fortified with more enzymatically stable citrus flavanones [117].

### 3.4. Terpenoids: Carotenoids

The major terpenoids in citrus fruits are carotenoids and limonoids, which are mainly found in peel and pulp. The color of citrus fruits is primarily due to their carotenoid pigments, which are tetraterpenes which exhibit yellow, orange, and red colors [125]. On the other hand, limonoids, which are oxygenated terpenoids, impart a distinctively bitter taste to the fruit. These terpenoids affect the color and taste of citrus species, which, in turn, influences the consumer acceptance. Due to their antioxidant activity, carotenoids have the potential to reduce the risk of cardiovascular disease, eye disease, as well as cancer, including skin cancer [126]. Several randomized controlled trials have investigated the effect of beta-carotene supplementation on cancer incidence, concluding that beta-carotene supplementation is not recommended as it has no beneficial effect on cancer prevention [127].

While nutritional supplementation with carotenoids to prevent heart and eye disease is commercially available, further in-depth studies are needed to define a preventive and therapeutic strategy. *Citrus* carotenoids, especially extracts of *C. reticulata*, also exhibit antimicrobial activity [128]. Nevertheless, the antimicrobial potential of citrus carotenoids needs further validation by in-vivo and clinical studies.

### 3.5. Vitamins

*Citrus* fruits are known to be rich in vitamin C, which is mainly contained in their peel. It has also been shown that citrus fruits contain significant amounts of vitamin A and B complex [129]. Some of the commercially available vitamin-C supplements and skin-care cosmetic products (such as anti-ageing creams and serums) are derived from citrus fruits.

### 3.6. Minerals

Calcium, potassium, sodium, magnesium, iron, copper, manganese and zinc have been reported to be present in citrus [129]. However, to the best of our knowledge, there is no commercially available nutraceutical or pharmaceutical containing any of the above minerals derived from citrus fruits.

### 3.7. Essential Oils

Essential oils are isolates of plant volatile metabolites containing various lipophilic substances. They are oily liquids with a strong odor, usually lightly colored and insoluble in water and have lower density than water. Essential oils are usually defined as extracts obtained by the distillation of different plant parts; however, in the case of citrus oils, they can be obtained by cold pressing from peel. Although conventional methods such as hydro-distillation, steam distillation, and cold pressing are most commonly used for the isolation of EOs (Table 1), novel techniques such as supercritical fluid extraction, microwave-assisted hydro-distillation and hydro-diffusion, etc., have also been used recently. The applied EOs isolation method has a great influence on the chemical profile of the EO, but factors such as species, harvest time and location, cultivation method, fruit storage conditions, climate, soil type, plant organs used, and plant vegetative cycle stage also play a role [130,131,132]. From the data presented in Table 1, it can be seen that the scientific literature usually reports the chemistry of essential oils of different citrus species (fruits, leaves, roots, and flowers, etc.) obtained by hydro-distillation using a Clevenger-type apparatus.

The yield of citrus-peel EO ranges from 0.5 to 5.0% (*w*/*v*) [130] and they are complex mixtures of chemical compounds from different classes, generally divided into the volatile fraction (85–99%) and the non-volatile compounds (2–6%, long-chain hydrocarbons, fatty acids, waxes, carotenoids, sterols, and coumarins, etc.). The volatile fraction is dominant in citrus EOs and consists mainly of monoterpenes, sesquiterpenes and their oxygenated derivatives, but other compounds such as aliphatic alcohols, aldehydes and esters can also be found [131,132].

Table 1 lists the main compounds detected in EOs of major citrus species from recent studies, and it can be seen that limonene, an aliphatic hydrocarbon (cyclic monoterpene), is the main component in citrus EOs. However, its content varied from sample to sample due to the influence of various parameters discussed above. Its content in lemon peels ranged from 40 to 86%, in orange peels from 46 to 95%, in lime peels from 13 to 77%, in tangerine peels from 55 to 88%, in grapefruit peels from 86 to 93%, and in pomelo peels from 54 to 97%. This compound was also found in other *Citrus* plant parts in appreciable amounts. Among others, γ-terpinene, β-myrcene, pinene, ocimene, linalool, α-terpinene, and sabinene were detected in most samples. *Citrus* essential oils are generally considered safe and are known for their numerous beneficial biological effects, such as anesthetic, sedative, analgesic, antimicrobial and anti-inflammatory activities, and are, therefore, used in pharmaceuticals; however, in addition, they have also been used for food and beverage flavoring, food packaging, and in different cosmetic products [133].

## 4. *Citrus* Uses in the Food Industry

Nowadays, consumers are more aware of the relationship between diet and health. In fact, the global consumption pattern has shifted toward foods that provide both nutritional value and health benefits. Therefore, the demand for functional foods has experienced an exponential growth in recent years [134].

*Citrus* fruits are excellent sources of natural bioactive compounds with well-known health-promoting properties. As previously described, the main citrus phytochemicals include polyphenols (mainly flavonoids), carotenoids, vitamins, organic acids, dietary fiber, and essential oils, which have promising biological activities due to their anti-oxidative, anti-inflammatory, and anti-carcinogenic properties [19]. These health benefits associated with citrus bioactive compounds are one of the main reasons the citrus-based food industry is expanding [134].

**Figure 1:**
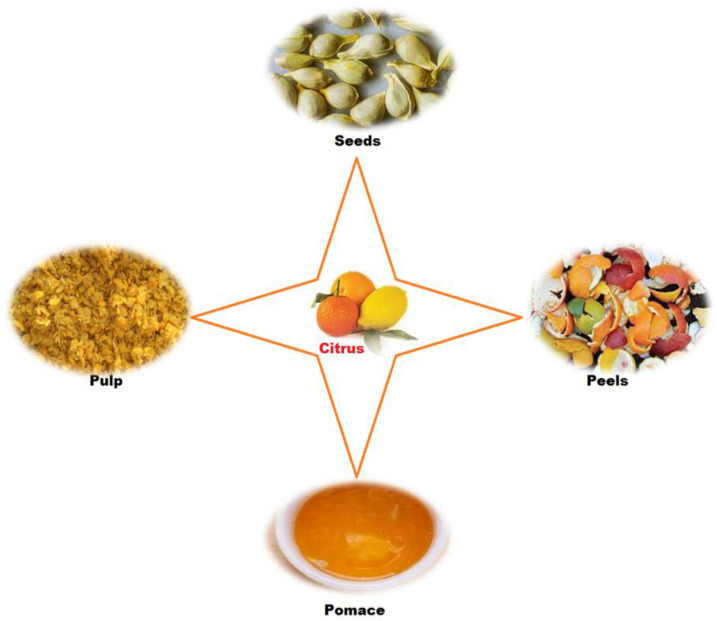
Citrus-fruit wastes: seeds, pulp, pomace and peels.

The mentioned citrus phytochemicals are mostly found in citrus-fruit wastes, namely, citrus peels, seeds, pomace, and pulp (Figure 1), which accounts for about 50 to 60% of the total weight of the fruit. Large amounts of processing waste are generated during the production of orange and other citrus juices, mainly the peel, cores, and segment membranes. OP is the main by-product, accounting for about half of the fruit mass. In fact, the citrus-processing industry generates more than 60 million tons of waste worldwide [18]. In the USA alone, juice processing of oranges and grapefruits generates more than 5 million tons of citrus waste [135] and 700,000 tons of OP annually [136]. In India, about 2.15 million tons of citrus peels out of 6.28 million tons of citrus fruits are generated from citrus juice processing annually.

**Figure 2:**
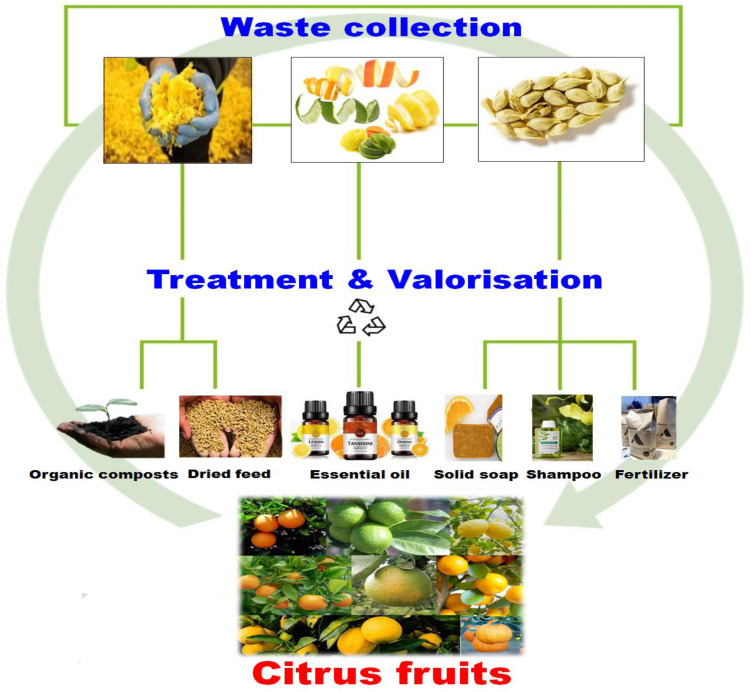
By-products created as a result of industrial processing of citrus-fruit wastes.

When OP goes unused, they become waste and are a possible cause of environmental pollution. Therefore, it is urgent to investigate and find solutions to convert these wastes into economically valuable products. OP were traditionally dried and marketed as livestock feed or used in the food industry [137] (Figure 2). They have been used to produce animal feed [11,138], single-cell proteins, fibers, pectinase/cellulose [139], immobilization support [140], ethanol, and bio-sorbents for heavy-metal removal [141]. Currently, the extraction of phenolic compounds from OP has attracted considerable scientific interest for use as natural antioxidants, especially in foods to prevent rancidity and the oxidation of lipids [142,143,144].

### 4.1. Extraction of Bioactive Compounds for Food Applications

In recent years, considerable attention has been paid to the extraction of bioactive compounds from citrus waste for further use as food additives, encapsulants, nanoparticles, prebiotics, or as a source of pectin, essential oils (Figure 2), polyphenols, carotenoids, or dietary fibers [145,146,147,148].

The extraction, isolation, and characterization of the mentioned bioactive components from citrus waste represent crucial steps in their recovery. Appropriate extraction conditions must be tested, optimized, and used to ensure that no degradation or loss of the bioactive compounds occurs. In fact, the solvent selection and extraction technique are the most crucial steps to maximize the yield of bioactive compounds [19,134]. Recently, several authors have focused their efforts on describing, in detail, the extraction techniques as well as the conditions employed to recover the different bioactive compounds from citrus wastes to improve the extraction yield [18,149,150]. Traditionally, the most commonly applied techniques include maceration, hot-water extraction, solvent extraction, and alkaline extraction [147,150]. Dar et al. [151] employed a maceration method to recover bioactive compounds from citrus peels, testing different solvent compositions (ethanol, water and 50% aqueous ethanol) for 8 h at room temperature. These authors reported that the extraction yield was higher with aqueous ethanol, followed by ethanol and water (29.28, 23.65 and 6.53%, respectively). In another study [152], phenolic compounds, ascorbic acid and the free radical scavenging activity of peel and pulp from Orlando orange, Kinnow mandarin and Eureka lemon fruits were assessed applying a conventional extraction method (80% aqueous ethanol at 70 °C in a shaking water bath for 3 h). These authors reported different levels of compounds in the matrices studied, demonstrating the major advantage from conventional extraction methods versus the most recent ones, namely, their versatile application in different matrices as well as their easy operation and low cost of application. The work developed by Gómez-Mejía and co-workers [153] is also in-line with these achievements. These authors tested the extraction of bioactive polyphenols from different citrus peels by employing magnetic stirring, evaluating the influence of extraction time (X1; 10–15 min), ethanol–water ratio (X2; from 20:80 to 40:60 *v*/*v*), and extraction temperature (X3; from 62 to 90 °C). Depending on the type of citrus peel, different extraction conditions were validated, but these authors concluded that the proposed conventional extraction method uses a lower amount of ethanol, reduced extraction times and a lower sample-to-solvent ratio than the novel techniques. Nonetheless, significant efforts have been made to replace these traditional techniques with green extraction methods, such as ultrasound-assisted extraction (UAE), subcritical water extraction (SWE), supercritical fluid extraction, microwave-assisted extraction (MAE), and enzyme-assisted extraction, not only to improve the extraction efficiency of bioactive compounds from citrus waste but also to overcome some of the most common drawbacks of traditional extraction techniques [145,146]. Šafranko et al. [146] investigated a two-step green extraction technique to recover bioactive compounds from mandarin peel (*Citrus unshiu* Marc. Var. *Kuno*). Firstly, these authors employed a supercritical CO2 (SC-CO2) extraction to recover volatile compounds, reporting limonene as the dominant component (30.65% at 300 bar), followed by farnesene, linoleic and hexadecanoic acids. Afterwards, the residue from SC-CO2 treatment was subjected to different SWE conditions to obtain bioflavonoids. Hesperidin (0.16–15.07 mg/g) was the most abundant flavanone in mandarin peel, followed by narirutin, and rutin. However, these authors reported that for extraction temperatures higher than 160 °C, the possible formation of undesirable compounds, such as chlorogenic acid and 5-hidroxymethilfurfural, can represent a limitation for the large-scale exploitation of the SWE technique. Another environmentally friendly extraction technique widely used is UAE [148]. Ordóñez-Santos et al. [148] employed UAE for the recovery of carotenoid compounds from mandarin epicarp to be further used as a natural colorant in bakery products. These authors reported a total carotenoid amount of 140.70 ± 2.66 mg β-carotene/100 g of dry sample for an extraction performed at 60 °C for 60 min. Montero-Calderon et al. [154] also employed a UAE technique to extract bioactive compounds from orange (*Citrus sinensis*) peel. At the optimal UAE conditions (50% aqueous ethanol, 30 min, 400 W of power), a total carotenoid concentration of 0.63 mg ß-carotene/100 g, vitamin C concentration of 53.78 mg AA/100 g, a total phenolic content of 105.96 mg GAE/100 g, and a hesperidin maximum concentration of 113.03 ± 0.08 mg/100 g were obtained. Despite the advances in the recovery of bioactive compounds from citrus matrices, most of the applied environmentally friendly extraction methods still cause concerns about the health and safety of the produced bioactive extracts, and the possible degradation and/or formation of undesirable compounds due to high temperatures [150]. Indeed, Benassi et al. [155] tested three different techniques, namely, conventional hot-water extraction, rapid solid–liquid dynamic (RSLD) and MAE, to recover pectin from waste orange peel. These authors concluded that the “hot-water” extraction assisted with citric acid was the most sustainable extraction route, ensuring higher extraction yield (21%) as well as a high quality of the extracted pectin (degree of esterification of 82.5%). This fact evidences the main advantage of the traditional extraction method—namely, its simplicity of operation and/or equipment—over the novel UAE, MAE or SWE, especially at an industrial scale. Therefore, researchers need to increase their efforts to find more sustainable, economical, and rapid techniques to recover the different bioactive compounds from citrus waste and to enable their safe incorporation into food products.

### 4.2. Food Industrial Applications

#### 4.2.1. Functional Food Ingredient

In recent years, consumer attention is moving towards consuming dietary-fiber-enriched foods. Considering that citrus fruits are excellent sources of antioxidants and dietary fibers, their inclusion in daily consumed foods such as baked goods, meat, and dairy products has become a hot topic of scientific research. Several authors have reported the potential of using citrus powder or flour in bakery and confectionery products as a functional ingredient [156,157,158]. For example, Caggia et al. [156] developed a low-fat bakery product (brioches) fortified with proportions (30, 50 and 70%) of debittered orange fibers, which improved the stability and nutritional properties of the developed product. The results obtained demonstrated that the addition of 50% debittered orange fiber resulted in a fat content of 4.5% in the products, in comparison to the 10% fat in the control sample.

Furthermore, due to the antimicrobial properties of these natural extracts, food product safety was also ensured. In another study [158], the positive effects of citrus albedo addition on bread shelf life due to the high pectin and fiber content was confirmed. The authors demonstrated that the partial replacement of wheat flour with dried fruit-peel powder provided a higher ability to bind large amounts of water. However, changes in the mechanism of staling, as well as structure modification as a consequence of fortification, should be further investigated. Iftikhar et al. [157] also demonstrated that *Citrus sinensis* (sweet orange) peel can be used to enhance the nutritional and functional properties of cakes due to their fiber and fat content. These authors concluded that the mixture of wheat flour with up to 3% citrus-peel flour is suitable for the development of cake with acceptable sensory attributes.

#### 4.2.2. Food Additive

Food additives are responsible for the flavor, color, taste, and nutritional qualities of food products. In recent years, with the increasing consumption of organic foods, the replacement of synthetic food additives with natural ones represents a great advantage in the field of food-processing industry [19]. In this sense, another reported application for the bioactive compounds with antioxidant activity recovered from citrus waste is their use as food additives, especially in the preparation of candied products for confectionery/baking industry [159,160]. For example, Romero-Lopez et al. [161] prepared muffins enriched with different proportions of dietary-fiber-rich orange bagasse and reported that the prepared muffins (with 15% extract) had a high dietary fiber (15.3%) and low fat (15%) content compared to the control muffins. Furthermore, the addition of the dietary-fiber-rich orange-bagasse extract to the muffin reduced the predicted glycemic index, and no difference in sensory evaluation was observed between the control muffin and the muffin prepared with dietary-fiber-rich orange-bagasse extract. These results are of the greatest interest because the addition of dietary-fiber-rich orange-bagasse extract to bakery products may be an alternative for people who require foods with low glycemic index. In another work, Ojha and Thapa [162] also prepared biscuits by replacing the wheat flour with mandarin-peel powder (3, 6, and 9%). They reported that biscuits formulated with 6% of mandarin-peel powder were comparable to the control biscuits with no substitution; the content of fiber, ash, ascorbic acid, carotenoids, polyphenol and antioxidant activity improved, and the reported values were 0.85%, 1.32%, 1.5 mg/100 g, 69 μg/g, 2150 μg gallic-acid equivalents/g and 24.5%, respectively.

Regarding the application of citrus extracts as food additives in another type of product, the research from Nishad et al. [159] should be highlighted, which investigated the potential of using citrus-peel extracts in the maintenance of oxidative stability of meat balls during frozen storage. The authors demonstrated that the natural antioxidant extracts from citrus peels can control lipid oxidation in meat products, by inhibiting enzymatic reactions responsible for oxidative damage. Moreover, the addition of citrus extract had a positive effect on the color, flavor, and overall sensory properties of the meat balls, indicating that it can be used as a natural preservative for foods rich in fatty acids. Younis et al. [163] also incorporated the mosambi peel, a by-product of the juice industry, in sausages and patties and reported an enhancement in fiber content as well as in fat and moisture content. In addition, the addition of up to 6% of mosambi-peel extract improved storage stability, demonstrating the potential of using citrus waste as a food additive in meat products.

#### 4.2.3. Food Colorant

The peels of citrus fruit are described as an excellent source of carotenoids. Not only do they impart color to fruits, these compounds also promote health benefits, which has attracted the attention of food industry as a solution to replace harmful synthetic colorants [134,147]. Barman et al. [164] used orange-peel waste to extract β-carotene, which was used to develop a stable nanoemulsion to be further employed as a natural colorant in food products. These authors reported that the addition of the nanoemulsion to fruit juice significantly enhances its color, thus providing an alternative to the use of synthetic colorants. Ordóñez-Santos et al. [148] optimized the process of ultrasound-assisted extraction of total carotenoids from the mandarin epicarp and demonstrated its potential to reduce the use of tartrazine in bakery products, such as cakes and bread, and the potential of its further use as a natural coloring additive. Ciriminna et al. [141] also investigated the technical and economic possibilities of using lemon-peel waste to produce water-soluble yellow colorant limocitrol 3-O-6″-[3-hydroxyl-3-methylglutaryl)])-d-glucopyranoside as a substitute for tartrazine. The authors demonstrated that this natural colorant can be easily obtained by simple solid–liquid extraction in aqueous ethanol or via hydrodynamic cavitation of lemon-peel waste in water.

Moreover, the obtained results on the chemical and physical stability of this natural colorant open the possibility to explore the industrialization of this new bioeconomy production. Despite these promising results, further research is needed to overcome the main limitations, such as the high cost of using natural biocolorants in industrial food applications [134].

#### 4.2.4. Flavoring Agent

Synthetic flavors are still widely used in the food industry; however, the use of citrus essential oils as flavoring agents is gaining increasing attention. Essential oils, mostly recovered from citrus peel, are prominent sources of terpenoids, which are widely used as flavoring agents in foods, and also have antibacterial, antifungal, and insecticidal properties [165]. Most studies have focused on determining the volatile profiles of different citrus species [166], and only few applications of citrus in the food industry as flavoring agents have been found. Bergamot oils, a rich source of linalool and linalyl acetate with promising flavor characteristics, have been used in some flour-based confectionery in recipes to replace bergamot peels [128]. Recently, Matsuo et al. [167] studied the effects of adding *Citrus natsudaidai* (CN) peel extracts to aqueous solutions and reported that the solutions flavored with CN extracts exhibited preferential odor over the commercial citrus-flavored beverages, which were classified in the same group as commercial citrus juices by the electronic nose test.

In addition, the solutions flavored with CN extracts exhibited sourness, bitterness, and an orange-like taste, and the overall acceptability was not significantly different from commercial citrus-flavored beverages. The use of citrus essential oils in ice cream, marmalade, and jam-like food products has also been widely described by other authors [166].

#### 4.2.5. Thickening Agent

As previously reported, citrus wastes, especially citrus peel, are an excellent source of pectin, which is extensively used in jams, jellies, marmalades, milk, and confectionery products due to their gelling and stabilizing properties [149]. Many studies have focused on finding more environmentally friendly extraction techniques to recover pectin from citrus peels [155,168], and very few papers reported results on its incorporation in food products. For example, Mann et al. [169] reported the production of ice cream using frozen Kinnow peel; both unblanched and blanched, at three levels (1, 3 and 5%). The addition of Kinnow peel improved the appearance, flavor, and overall acceptability of the ice-cream samples. The authors reported that the content of ascorbic acid and flavonoids (namely, naringin) in the ice-cream samples increased with the addition of Kinnow peel, showing that the best levels of frozen Kinnow peel, based on sensory evaluation, were unblanched—3% and blanched—5%. Mohamed et al. [86] reported the extraction of pectin from white and red Sudanese-grapefruit peel and confirmed that the gel-forming quality of the extracted pectin was similar to that of commercial pectin.

Jellies prepared with both types of grapefruit peel pectin set within the 10–25 min, indicating them to be rapid-set pectin and demonstrating their potential to be used as a stabilizer/thickening agent in different food products. In another study [170], jams were also prepared and their physicochemical and sensory properties analyzed. The authors extracted, characterized, and applied pectin recovered from grapefruit peel from Duncan cultivar to jam formulations and observed a significant effect on the texture of the final product. Despite the limited number of studies demonstrating the practical applications of extracted pectin in food products, this research has great potential, as the extracted pectin from citrus waste can replace the use of commercial pectin as a gelling agent in various foods.

### 4.3. Limitations of Applying Citrus Wastes in Food Industry

Several studies have demonstrated the promising potential of incorporating citrus-waste extracts in food-industry products. However, some parameters of citrus extracts, such as their low stability and water solubility, limit their further use at a larger scale. Since most of the bioactive compounds present in citrus extracts have poor bioavailability and increased sensitivity to different environmental conditions, such as pH, heat, and oxidation, their protection is a major challenge for the food industry in commercial applications.

In addition to these limitations, the conversion of citrus wastes into value-added food products raises concerns about the safety and toxicity of the citrus-waste extracts used [171]. In general, the potential of citrus wastes to be used as novel functional ingredients with a specific function is well-described; however, the evaluation of their safety has not yet been established. Nevertheless, the use of citrus wastes in food products must comply with current legislation and a risk assessment must be performed to assess their safeness, and very few studies have been conducted recently to address these issues [156,172]. Therefore, a holistic research approach is needed to integrate the value-addition strategy with risk analysis and to apply forecasting and optimization studies to the whole supply chain.

Furthermore, industrial-scale studies on the use of citrus food are still very limited, although they are also extremely necessary to define the barriers to a large-scale application. Therefore, collaboration between academic and industrial partners may be the key to increase the value of citrus-processing industries by converting their wastes into functional food products.

### 4.4. Application in Food Packaging

According to definition reported in the EC Regulation No 450/2009, “active materials and articles means materials and articles that are intended to extend the shelf-life or to maintain or improve the condition of packaged food; they are designed to deliberately incorporate components that would release or absorb substances into or from the packaged food or the environment surrounding the food”.

Active packaging technology provides several advantages over the direct addition of active compounds to the packed food, such as the lower amounts of active substances required, the localization of activity at the surface, migration from the film into the food matrix, controlled release systems, and the elimination of additional steps within a standard process intended for introducing the active compounds at the industrial processing level, such as mixing, immersion, or spraying. Controlled-release systems are of industrial importance as they can prevent sensory or toxicological problems or inefficiencies of the system caused by too-high or too-low concentrations of the delivered substance [173].

In the review by Han et al. [174], the problems related to development of antioxidant and antimicrobial active packaging are well-defined, making it quite difficult to set specific targets for the selection of the natural extracts to be used due to the absence of reference benchmarking products. Recent research trends have focused on the development of active food packaging by adding antioxidants into packaging materials to extend the shelf life of the product. The most commonly used synthetic antioxidants in the food industry are butylated hydroxyanisole (BHA), butylated hydroxytoluene (BHT) and propyl gallate in U.S.A., especially for packaging cereals and snacks [175]. However, synthetic antioxidants can also be carcinogenic and harmful to consumers. This must be considered in active packaging, as migration from food contact materials is not negligible and is, indeed, a desired phenomenon. With increasing health awareness and consumer’s demand for reduced use of chemicals in food packaging, more attention has been paid to finding naturally occurring, safe substances that can act as alternative antimicrobials and antioxidants. The use of natural antioxidants derived from plant extracts in food packaging is becoming increasingly popular.

Extrusion is the most popular technique to include natural extracts into the final formulation [173]. In this technique, bioactive compounds are incorporated before extrusion, so that the high temperatures of extrusion (the exact values depend on the melting temperature of the processed polymer) allow their effective and homogeneous distribution in the film, although, at the same time, they are responsible for the thermal degradation of the bioactives’ activity. For example, Ha et al. [176] used a high-temperature profile (160–190 °C) to extrude an antimicrobial LLDPE-based film, which resulted in a high loss of functionality of the grapefruit-seed extract (GSE) and loss of antimicrobial activity. For this reason, heat-sensitive bioactive agents (i.e., natural extracts) should preferably be incorporated into the packaging using non-heating methods (e.g., electrospinning and surface coating). Among these methods, surface coating, in particular, is a simple process that relies on low temperatures but may suffer from poor adhesion to plastics and needs to be designed to be in direct contact with the food in cases where active packaging is the final objective of material production.

Natural extracts are already produced and commercialized by different companies, mainly for direct use in food or for the cosmetic and pharmaceutical industries. There are numerous scientific articles on the incorporation of such natural extracts to extend the shelf life of food products. However, large-scale demonstration is still pending [177], especially in relation to their use as packaging materials. While synthetic antioxidants are generally added to improve the properties of the materials during processing, natural antioxidants suffer from the major drawback of thermal degradation at the typical working temperature of the extrusion processes [178]. Therefore, encapsulation of antioxidants can improve their thermal resistance so that they can be incorporated directly into the plastic pellets before extrusion.

Green tea, rosemary extracts, essential oils and various fruit extracts are the most-used antimicrobial and antioxidant products investigated in the literature for packaging applications [179,180,181,182,183]. In 2015, Goglio Spa (Italy) won the Packaging Oscar for its product GTea^®^, an active packaging with a green-tea extract.

As the importance of environmental sustainability and circular economy is increasingly recognized, it would be better to use extracts obtained from agri-food residues, such as from orange peels.

#### 4.4.1. Natural Extracts Requirements for Incorporation into Packaging Material

A key point in selecting the extract to be incorporated in active packaging is obviously the food-grade characteristic. It would be the best to use food-grade extracts since there will be no problem with migration restrictions, especially if active packaging is planned with the expected release of extracts into food. On the other hand, if the extract is not food-grade, it will have to be exploited to absorb substances from the packaged food or the environment surrounding the food and then incorporated only into the external coating, or into an intermediate layer (i.e., by incorporation into an adhesive if lamination is used to manufacture the multilayer film) with a barrier layer which prevents migration into the food.

If the aim is to provide antioxidant and/or antimicrobial activities, these properties are crucial for selection and it is necessary to verify the maintenance of the property after incorporation and over time. Furthermore, depending on the selected target food to be packed and the coloring power of the extract, this property could be incompatible with obtaining a suitable transparent packaging. However, it should be noted that many natural extracts, such as those from citrus species, have antioxidant/antimicrobial activity due to the presence of phenolic compounds, which are often colored. At the same time, the presence of phenolic compounds and carotenoids with the ability to absorb light in the 200–800 nm range could be of interest, as UV-Vis light can catalyze many degradation reactions in food products.

#### 4.4.2. Need for Encapsulation

As explained earlier, natural extracts are sensitive molecules that can be denatured under harsh conditions. Encapsulation may be necessary to provide suitable solubility in the coating medium (when incorporated into the packaging material via a coating application), thermal stability at processing temperatures (when incorporated into a plastic polymer prior extrusion step), and/or light stability.

The thermal stability of natural additives is the main problem in cases of the direct incorporation of the extract for compounding a functionalized polymer masterbatch. The working temperature during extrusion to form the plastic film is the most challenging point, since it can exceed 150–200 °C depending on the processed polymer. For this reason, direct incorporation prior to film extrusion is often discarded. Spray-drying encapsulation can be applied to increase the thermal stability of the extracts. Thermal stability may also be required for some specific uses of packaging in the food industry, such as hot filling and thermal treatment after packing. In addition, other encapsulation technologies may be considered: extrusion with vibrating nozzles, jet cutter, coacervation and others.

Information on the maximum temperature that could be reached during the coating preparation or during the melting/extrusion process of the plastic material (in the case of direct incorporation of the extract into the polymer), or, eventually, by the final food industry end user, is, therefore, key to defining thermal-stability requirements.

Solubility in water or in another solvent is necessary if the extracts are to be incorporated via a coating application, depending on the solvent on which the coating is based. Furthermore, in Europe, the legislation for plastic food-contact materials (FCM) reports the use of different simulants to simulate the different ranges of food products (ethanol 10%, 20%, 50%; acetic acid 3%; vegetable oil with less than 1% unsaponifiable content, and simulant E for dry foods). Evaluation of solubility in these simulants is important in terms of desired or undesired release in the packaged food.

Encapsulation may affect and eventually improve thermal stability and solvent solubility, depending on the carrier materials used, but it is also important to check the potential effect of the encapsulation process on the antioxidant and antimicrobial properties of the original extract.

#### 4.4.3. Literature Examples of Citrus Extracts Use to Develop Antimicrobial/Antioxidant Packaging

Plant/fruit extracts or essential oils are known for their potential antioxidant and antimicrobial properties and have been widely investigated in the literature for these properties as well as for their use in active food packaging [184]. Many of the extracts studied are obtained from fresh plants, fruits and herbs, but not from processing residues.

The antibacterial capacity of 32 essential oils against five foodborne (*L. monocytogenes*, *S. aureus*, *E. coli*, *S. Typhimurium*, *P. aeruginosa*) and spoilage bacteria in liquid phase (as minimum inhibitory concentration, MIC, values) was evaluated by Ghabraie et al. [185]. Among the oils tested, Chinese cinnamon, cinnamon bark and wild-bergamot essential oils were the only ones that exhibited inhibitory activity against all five pathogenic microorganisms tested.

However, these essential oils were not produced from residues and by-products, like in the case of essential oils from fruit peels. In addition, essential oils have a typical strong flavor that may interfere with their use in food packaging, as they could have a strong impact on the sensory profile of the packed foods.

Grapefruit-seed extract is made from the seeds and pulp of grapefruit and it contains tocopherol, citric and ascorbic acids [186]. The antioxidant and antimicrobial effects of this extract have been reported in different products such as ground beef [186]. There are several studies in the literature in which grapefruit-seed extracts were incorporated into bio-packaging [76,180], which showed good antimicrobial activity against *L. monocytogenes* and *E. coli*.

Kanmani and Rhim [187] incorporated a GSE at different concentrations (from 0.6 to 13.3 μg/mL) into an agar-based film through a casting technique and the obtained films were evaluated for their antimicrobial activity against *L. monocytogenes*, *Bacillus cereus*, and *E. coli*. Only the enriched films showed antimicrobial activity, with an extract-dose correlation, and better performance against *L. monocytogenes* (Gram +) than against *E. coli* (Gram -), probably due to the specific outer membrane which inhibits the diffusion of the active compounds through the lipopolysaccharide layer.

Extracts can be obtained from citrus peel, which is a residue of citrus-juice production. Jridi et al. [188] incorporated phenolic extracts from red-orange peels (*C. sinensis*) in both dried and fresh forms at concentrations of 5 and 10 mg/mL into a fish gelatin-based film using the casting technique. The films were tested for their antimicrobial activity against *Micrococcus luteus*, *Staphylococcus aureus*, *Bacillus cereus*, *Pseudomonas aeruginosa*, *Salmonella enterica*, *Listeria monocytogenes* and *Enterobacter* sp. Some antimicrobial activity was exhibited against all the tested microorganisms, with *S. aureus* being the most sensitive. In general, the fresh extract was more effective than the dry extracts, showing a reduction in activity after drying.

The focus of the study of Bassani et al. [189] was the development of an innovative, biodegradable, and sustainable PLA-based active-packaging solution incorporating an antioxidant extract from orange peel. The extract was first obtained using hydro-alcoholic extraction and then purified by a resin absorption process (which was required to remove sugars and organic acids and increase antimicrobial activity). The extract contained up to 50% total phenols on dry matter and it was either freeze-dried or spray-dried with pectin or ß-cyclodextrins as a carrier material to obtain powder formulations. Encapsulation could improve thermal stability compared to the freeze-dried extract, particularly when cyclodextrins were used. The three powder extracts were incorporated into commercial PLA at different concentrations (0.25, 0.50, 1.50, 2.0 wt %.) and film samples were obtained by hot pressing. The films were assayed to evaluate the influence of the extract addition on the thermal stability of the polymer, color, and mechanical properties of the films under accelerated light storage conditions (in a Suntest XXL+ aging chamber, for 500 h). Positively, extract addition preserved the transparency of the bioplastic and did not modify the degradation temperature profile of the PLA film. However, the extracts resulted in a yellowish coloration that increased with the amount added and achieved an unacceptable browning at a dosage of 2%. The accelerated light storage test highlighted that encapsulation improved color stability and that the film performed worse in terms of mechanical properties (Young module, tensile strength and elongation at break) when it was enriched with the extracts (in this case, the freeze-dried formulation was preferable to the encapsulated ones).

In the work of Fiorentini et al. [190], different commercial citrus-peel extracts were investigated for their thermal stability, which was then improved by a spray-drying encapsulation process with beta-cyclodextrins. The study revealed that the antioxidant capacity was retained after the encapsulation process, with an apparent 20–25% reduction in the total phenolic content of the original extract. In addition, the antimicrobial activity against *S. aureus* was almost unaffected by spray drying, with MICs ranging from 5–0.625 mg/mL to 5–1.25 mg/mL. The encapsulated extract with the best antioxidant and antimicrobial activity was incorporated into a polylactic acid/polyhydroxy butyrate (PLA/PHB) film produced on an industrial scale by cast extrusion. The obtained extract-enriched film was proven to be compliant with European regulations for food-contact materials in relation to overall migration in contact with acidic, hydrophilic and fat-containing food categories. The authors also evaluated the migration of active compounds and observed a potential release of 13.41% in hydrophilic food products and 11.02% in acidic products (pH < 4.5). The film showed growth inhibition by 30 and 60% against *E. coli* and *S. aureus*, respectively.

**Figure 3:**
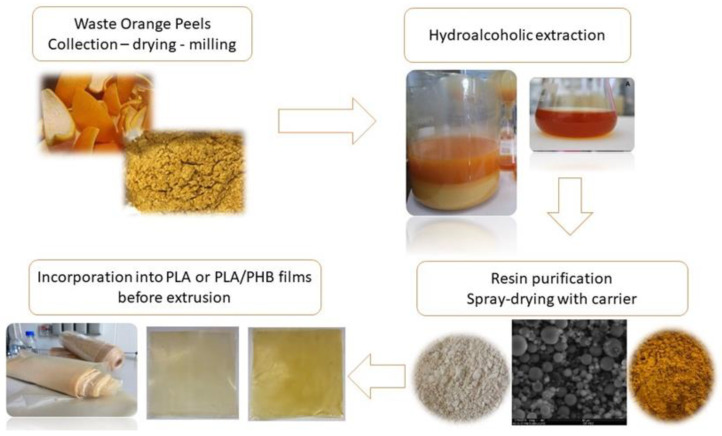
Schematization of the process from waste citrus peels to purified spray-dried extracts (powders obtained with β-cyclodextrins on the right or pectins on the left, at different extract-to-carrier ratios, and example of a SEM observation), and to their incorporation into PLA (on the right) or PLA/PHB (on the left) films.

The main steps applied in the works [189,190] for the production of antioxidant/antimicrobial extracts and their incorporation into biobased plastic materials, are summarized in Figure 3. It is clear how different encapsulation processes and percentages of addition may lead to more or less colored final materials.

Colon and Nerin [191] tested a grapefruit dried hydroalcoholic extract for incorporation into a coating applied on a PET film. The system was prepared according to EU patent EP1477519-A1.41 and the coating was applied at room temperature reaching a maximum of 40 °C using hot air to eliminate the solvent. Active films were prepared with different weight percentages of the active agent/active layer, which varied from 0.7 g active/m^2^ film to 3.0 g active/m^2^ film. The obtained films were subjected to a free radical gas stream to increase oxidation and the antioxidant capacity was then determined based on the oxygen radical absorbance capacity (ORAC) assay. The grapefruit extracts revealed a lower antioxidant activity (based on ORAC assay) compared to green-tea or green-coffee extracts.

A commercial grapefruit-seed extract (GSE) was also used by Wang et al. [76] to develop active films based on poly(lactide) (PLA) or antimicrobial low-density polyethylene (LDPE). In this study, the natural extract was added before the extrusion step. Thermoplasticized starch was used as a carrier to introduce the extract into the polymer. It was prepared by mixing corn starch with 20% wt glycerol (as plasticizer) and 40% of GSE and heating at 120 °C for 30 min in an autoclave. The plasticized starch mixture was then cooled to room temperature and pulverized using a blender. The obtained powder was mixed with the plastic resins (PLA and LDPE) at the ratio of 1:10 *w*/*w* to obtain the masterbatches, which were cast and blow extruded, respectively. The addition of the extract decreased the lightness of the films and increased the color (increase in the chromatic coordinates a* and b*) due to both the dextrinization of the starch and the phenolic content of GSE. The starch blend addition increased the LDPE thickness but not the PLA film. This is due to the fact that the hydrophilic thermoplastic starch is more compatible with more hydrophilic polymers, such as PLA. The antimicrobial activity of the films was tested against *L. monocytogenes* and *E. coli* and was high for the enriched PLA film, but not for the LDPE. Finally, the capacity of the films to act as active food packaging was tested on a fish paste, where the activity of the GSE-PLA film was confirmed, while the GSE-LDPE showed inhibitory activity only after 6 days of contact. This was consistent with the results of the migration tests, which showed a slower release of the GSE components from LDPE than from PLA.

## 5. The Use of Citruses in Cosmetics

The global growth and development of the cosmetics market is a well-recognized trend worldwide, accounting for 41% in 2021. It is estimated that at an annual growth by 6%, the value of the cosmetics market will reach USD 675 billion by 2026 (Expert Market Research). The cosmetics industry follows a continuous research and development (R&D) program, not only studying consumer behaviors and changes in beauty preferences, but also focusing on new technologies and sustainable development. Among all cosmetics categories (facial care, body care, hair care, sun care, decorative/beauty cosmetics, fragrances), skin-care products account for the largest market share, with trending demands in anti-aging and organic products with natural ingredients. It is estimated that the total expenditure on R&D in the cosmetics industry is EUR 2.35 billion, with an average of 5 years spent on innovative research and formulation to bring a new product to market (expert market research).

Before launching a new cosmetics product on the market, the safety of the product and compliance with regulatory requirements must be confirmed. This is most directly linked to the specific ingredients of the final formulation, as well as the packaging material. Before a new product is launched, the microbiological safety and efficacy of the new cosmetic formulation must be evaluated and confirmed through standardized testing. It is imperative to demonstrate in controlled studies that the new cosmetic product provides the claimed benefits to consumers. Subsequent stability tests provide information on the compatibility of the product and specific packaging, defining the products’ shelf-life. After all laboratory testing is complete, the process development scales up the technology, assuring the maintenance of the product quality. Finally, it is important to establish a supply chain which includes raw materials, packaging, and labeling to assure sustainability in the production, independently of the market demand.

Clear trends that can be recognized in the cosmetics industry are related to the inclusion of safe and environmentally friendly ingredients and technologies that respond to ever-changing consumer expectations. Increasing consumer awareness of the side effects of synthetic cosmetic ingredients and the advantages of natural and organic cosmetics, which generally produce fewer allergic responses and unwanted or unexpected effects, are leading to a shift in consumers’ demands for products in which the key active ingredients are of natural origin, most often plant-derived. The increasing demand for natural and organic products and attractive marketing strategies in this field has propelled the expansion of a wide range of new products containing various natural extracts and phytochemicals. In this respect, citrus fruits, as well as the by-products of their processing, represent very attractive alternatives for the reformulation of cosmetic products, or the design of completely new products with additional benefits. Citrus fruits are rich in numerous phytochemicals and bioactive components, representing excellent sources of antioxidants, polyphenols (mainly flavonoids), carotenoids, vitamin C, folic acid, minerals, and pectin [99]. Among flavanones, naringin and hesperidin have demonstrated numerous bioactivities that may be useful for the cosmetics industry, such as anti-oxidant, anti-inflammatory, and anti-carcinogenic properties [192], and they are mainly found in citrus peel and albedo. In addition to the use of citrus processing by-products to extracts’ added-value compounds, citrus essential oils are often incorporated as fragrance components due to their specific, unique fragrance and compatibility with cosmetic products [193].

Considering the worldwide mass production and processing of citrus, and the global industrial framework oriented towards circular economy and a sustainable approach in biowaste valorization, the recovery of bioactive ingredients and essential oils from citrus bio-waste is a current trend in the R&D sector of the cosmetic industry (Figure 2).

### 5.1. The Extraction of Cosmeceuticals from Citrus Biowaste

Citrus fruits are cultivated mostly in tropical and subtropical regions, with multimillion tons of annual production, generating approximately 40 million tons of citruses waste [4], which can be used to produce valuable compounds with potential uses in the food, nutraceutical, pharmaceutical and cosmetic sectors. In the food sector, citruses are mostly processed into juices and marmalades, generating large amounts of waste consisting of peels, pomace, seeds, and membrane residues. Citrus-peel waste accounts for 50–70% of processing waste and can be used for the production of flavoring agents, flavonoids, and citric acid for the cosmetic and pharmaceutical industries.

Strict legislative requirements for cosmetic products in terms of their safe use and residues of unwanted contaminants, such as traces of organic solvents or heavy metals, have made “green” techniques for the isolation of cosmeceuticals from citrus-fruit waste mandatory. An array of bioactive compounds with beneficial properties for skin care (such as anti-radical, antioxidant, whitening, and anti-inflammatory, etc.), and belonging to different chemical classes, can be isolated using modern extraction techniques with improved performance, lower energy and time consumption and reduced use of organic solvents. For example, carotenoids, which are concentrated in citrus peels and are responsible for the orange, yellow and red color of the fruits, have been extracted using ultrasound-assisted extraction, using different solvents, such as ionic liquids [194], ethanol [154] or limonene [195]. In addition, carotenoids can also be extracted using supercritical carbon-dioxide [196], or, as an advanced approach, high-voltage electric discharge (HVED) technology [197]. On the other hand, the essential oils of different citrus fruits (e.g., mandarin, orange, grapefruit, lemon, lime, kinnow mandarin) can be extracted from different parts of the plant, representing valuable natural cosmetic fragrances with distinctive characteristics and beneficial properties. The essential-oil yield obtained by microwave-assisted hydro-distillation from the peels of different citrus varieties can vary from 0.42% (*Citrus sinensis* L., orange) [198] to 2.73% (*C. sinensis* var. Valencia, mandarin) [199]. The yields obtained by supercritical carbon-dioxide extraction are much higher, reaching ~28% in citron (*C. medica*) peel [200]. Garrido et al. [201] isolated essential oils from lime and orange seeds with hexane assisted by ultrasound and achieved yields of 22%.

Other classes of compounds of interest to the cosmetic industry from an added-property point of view include phenolics, which can be extracted from virtually all citrus wastes, and all the green technologies listed. For example, total phenolics extracted with 20% glycerol from grapefruit peels by high-voltage electrical discharge was 1880 mg GAE/100 g [202], while with high hydrostatic-pressure technology applying 300 MPa, 266.23 mg GAE/100 g was extracted from lemon peel, 397.21 mg GAE/100 g from lime peel, 587.28 mg GAE/100 g from tangerine peel, and 288.16 mg GAE/100 g from orange peel, respectively [203]. The extraction of mandarin peels (*Citrus inshiu*) by microwave-assisted extraction with 70% ethanol resulted in the recovery of 5860 mg/100 g of hesperidin and 1310 mg/100 g of narirutin [204]. Depending on the solvent used, ultrasound-assisted extraction leads to similar yields of phenolic compounds as microwave-assisted extraction. On the other hand, supercritical carbon-dioxide extraction, even at a high concentration of co-solvent (40% ethanol), generally produce lower yields (0.67 mg GAE/100 g in grapefruit peels, 0.66 mg GAE/100 g in lemon peels, 0.45 mg GAE/100 g in orange peels, and 0.38 mg GAE/100 g in tangerine peels) [205].

### 5.2. The Use of Citrus Biowaste Extracts in Cosmetics

The bioactivity of different citrus-waste extracts has been undoubtedly confirmed by numerous studies, but real applications in the cosmetic industry are still scarce; thus, in-depth research in this field is needed. The next steps in the implementation of citrus waste in the cosmetic industry must be oriented towards the development of formulations integrating safe doses of extracts as well as studies of the quality parameters of such products, including basic physico-chemical quality parameters, efficacy, stability tests, and, no less important, consumer acceptance.

Kim et al. [206] confirmed several beneficial activities of ethanolic extracts of *Citrus unshiu* waste. In addition to antioxidant effects and dose-dependent inhibition of melanin synthesis, the extracts also showed moderate antibacterial effects against bacteria associated with acne, namely, *Propionibacterium acnes*, while they were ineffective against normal skin microflora *Staphylococcus epidermidis*. The absence of toxicity in human keratocytes was confirmed at concentrations below 10 μg/mL, resulting in 100% viability, while increasing the concentration to 100 μg/mL reduced the viability to 80%.

The extracts of obtained from *C. reticulata* Blanco peel showed promising anti-collagenase and anti-elastase activities which could be exploited for the formulation of anti-aging cosmetic products [207]. The authors compared the activity of extracts obtained using Soxhlet extraction and maceration and found better activities for hot extraction. The EC_50_ values calculated for inhibition of collagenase and elastase, for hot extraction and maceration were 329.33 μg/mL, 466.93 μg/mL, 3.22 mg/mL and 5.09 mg/mL, respectively. The authors also confirmed high anti-radical activities against 1,1-diphenyl-2-picrylhydrazyl (DPPH), superoxide anion, and 2, 2′-azino-bis (3-ethylbenzothiazoline-6-sulfonic acid) (ABTS) radicals.

Skin-whitening cosmetic formulations have significantly increased in popularity in some countries due to different beauty perceptions. In some cultures, and modern societies, pale skin is considered aesthetic, giving a rise to numerous skin-whitening products, such as soaps, tonics, and creams, etc. Some of these products contain dangerous synthetic chemicals, such as hydroquinone, or pure natural isolated compounds with anti-tyrosinase activity, such as kojic acid, alpha and beta arbutin, or the isoflavone glabridin, isolated from licorice root. Although they are all of natural origin, some of these phytochemicals have some safety concerns, such as kojic acid, which can trigger contact dermatitis [208].

### 5.3. The Use of Citrus Essential Oils in Cosmetics

The essential oils from citruses can be produced by steam distillation from different parts of the plant, mostly from peels, but also from flowers, shoots, buds, and leaves. Due to their very pleasant fragrance and antibacterial effects, citrus essential oils have been used in folk medicine and perfumery for centuries and are now popular ingredients in aromatherapy, air fresheners, house cleaning products, and cosmetics. Although citrus essential oils have a number of beneficial effects in skin care, such as degreasing, antiseptic, brightening and astringent properties, the contents of particular essential-oil components are regulated by legislation for cosmetic products due to potential adverse effects they might exhibit, such as phototoxicity or allergenicity. The phototoxicity of citrus essential oils and extracts may be related to furocoumarins, which strongly absorb UV light and carry a risk of skin burns, inflammation and swelling. Identified major phototoxins in citruses include bergamottin, citropten, herniarin or oxypeucedanin, bergapten and other derivatives [130]. Due to its recognized phototoxicity, the Scientific Committee on Cosmetic Products (SCCS) has implemented a restriction on furocoumarin-like substances in cosmetics, limiting their content to 1 ppm (SCCNFP, 2001). However, furocoumarins may have other medicinal applications in different skin conditions. The intense UV-light absorption of furocoumarins is the underlying mechanism for psoralen UV A (PUVA) treatment of psoriasis or vitiligo. In topical psoriatic plaques, psoralens form adducts with cellular DNA that induce apoptosis upon exposure to UV light. In this treatment, furocoumarins are applied either orally or topically before UV treatment. In topical application, the limiting step is weak percutaneous permeability, and in both cases, there is a risk for the development of cancer with long-term therapy [209].

The antimicrobial effects of citrus essential oils are beneficial in different skin inflammation conditions, such as acne, which is attributed to different mono- and sesquiterpenoids, aldehydes, ketones, acids, alcohols, and esters, such as p-cymene, limonene, carvacrol, geraniol, eugenol, and others, which can be well dermally absorbed due to their small size and hydrophobic characteristics. The peel of citrus fruits yields about 0.5–5% (*w*/*v*) essential oil, which is mainly composed of volatiles (85–95%) and non-volatile fractions consisting of coumarins, fatty acids, sterols, carotenoids and polymethoxylated flavonoids [130].

Many components of citrus essential oils act as efficient free radical scavengers, important for anti-ageing processes but also involved in melanogenesis via the activation of a melanin-producing enzyme—tyrosinase. It has been confirmed that essential oils of different citruses act as efficient tyrosinase inhibitors [210], and are, therefore, frequently added to cosmetic products as whitening compounds.

### 5.4. Cosmetic Formulations with Components Isolated from Citruses

Data on cosmetic formulations containing citrus extracts or oils are rather scarce in the literature; however, several formulations based on peels’ extracts were reported. The authors mostly emphasized the antiradical activity of peels extracts, confirming them using standardized spectrophotometric assays, such as the DPPH test. Kamalambigeswari et al. [211], for example, used the methanolic extract of lemon peel, while Riski et al. [212] prepared individual and combined 70% ethanolic extract from the peel of several citrus species, namely, lime fruit (*C. aurantifolia* Swingle.), kaffir lime (*C. hystrix* DC), and sweet orange (*C. sinensis* L.), to formulate creams with different extract concentrations (1–3%). The formulations met all quality and stability criteria for this product.

It is even rarer to find scientific publications on cosmetic formulations incorporating citrus essential oils, probably due to their photosensitivity and allergenicity. However, Nareswari and Kuncoro [213] extracted essential oil from lime (*Citrus amblycarpa*) leaves yielding 0.47%, and prepared an ointment with antibacterial properties. The principal stability parameters (pH, dispersiveness, density, homogeneity, sensory, viscosity, and adhesion) were evaluated after 8 weeks and found to be satisfactory. Another example of essential-oil addition for the purpose of additional activity was the formulation of a cream with mosquito-repellent activity with the addition of a lemon-grass (*Cymbopogon citrus*) essential oil which resembles citrus in fragrance [214]. The mosquito-repellent activity was confirmed in vivo in volunteers exposed to three mosquito species, namely, *Aedes aegypti*, *Anopheles stephensi* and *Culex quinquefasciatus*, for a duration of 240 min, in comparison to commercial herbal creams that were used as control.

Recognizing that citrus essential oils are prone to photo-sensitive degradation and volatility, Kaur et al. [135] formulated a nanoemulsion of *C. limonum* essential oil for further use in cosmetics. The authors used Tween 80 and ethanol as surfactant and co-surfactant, respectively, to form an oil-in-water emulsion with a particle size of 60 nm, a polydispersity index of 0.125 and a zeta potential of −14.9 mV. The antioxidant potential, chemical profile, and thermodynamic stability of the plain essential oil and the nanoemulsion-protected essential oil were monitored over a period of 6 months. The developed nanoemulsion proved to be much more stable; thus, its use in cosmetic formulations is recommended. For recognized anti-inflammatory and antibacterial properties, some authors reported that bergamot essential oil (*C. bergamia*) can be useful in antidandruff and hair-growth products [215]. The California Navel orange-peel extract (*C. sinesis* L.), due to its high anti-tyrosinase activity (IC_50_ = 255.10 μg/mL), was used for facial-cream formulation [216]. The facial cream was formulated with 2% (*w*/*w*) orange-peel extract and was evaluated as pleasant by a sensory analysis panel. The formulated product reduced melanin pigment by 17.33% after one month of use, without a single case of skin irritation reported in 20 volunteers [216].

## 6. Conclusions

This review provided an overview of the chemical constituents of the main *citrus* species and a comprehensive assessment of the health benefits of their consumption, with reference to various secondary metabolites such as flavonoids and terpenoids. The well-known nutraceutical and medicinal value of *citrus* fruits guarantees a protective effect against a series of chronic diseases, partly linked to the high content of nutrients with antioxidant capacity. Therefore, thanks to their innumerable beneficial properties, due to the synergistic action of their bioactive constituents, *citrus* fruits can be classified as functional foods.

Furthermore, in this review, aspects related to the use of *citrus* fruits and their extraction products such as essential oils, in the food and cosmetic industry sectors, also highlighting the latest technological developments, were taken into consideration, thus showing the high potential of *citrus* fruits as a source of multifunctional natural agents.

## Data Availability

Not applicable.
